# Radiomics for lung adenocarcinoma manifesting as pure ground-glass nodules: invasive prediction

**DOI:** 10.1007/s00330-020-06776-y

**Published:** 2020-03-11

**Authors:** Yingli Sun, Cheng Li, Liang Jin, Pan Gao, Wei Zhao, Weiling Ma, Mingyu Tan, Weilan Wu, Shaofeng Duan, Yuqing Shan, Ming Li

**Affiliations:** 1 Department of Radiology, Huadong Hospital Affiliated to Fudan University, Shanghai, 200040 China; 2 Diagnosis and Treatment Center of Small Lung Nodules, Huadong Hospital, Shanghai, China; 3 GE Healthcare, Shanghai, 210000 China; 4 Department of Radiology, The People’s Hospital of Rizhao, Rizhao City, 276800 China; 5 Institute of Functional and Molecular Medical Imaging, Fudan University, Shanghai, China

**Keywords:** Adenocarcinoma, Lung, Nomograms, X-ray computed tomography, Solitary pulmonary nodule

## Abstract

**Objectives:**

To investigate the value of radiomics based on CT imaging in predicting invasive adenocarcinoma manifesting as pure ground-glass nodules (pGGNs).

**Methods:**

This study enrolled 395 pGGNs with histopathology-confirmed benign nodules or adenocarcinoma. A total of 396 radiomic features were extracted from each labeled nodule. A Rad-score was constructed with the least absolute shrinkage and selection operator (LASSO) in the training set. Multivariate logistic regression analysis was conducted to establish the radiographic model and the combined radiographic–radiomics model. The predictive performance was validated by receiver operating characteristic (ROC) curve. Based on the multivariate logistic regression analysis, an individual prediction nomogram was developed and the clinical utility was assessed.

**Results:**

Five radiomic features and four radiographic features were selected for predicting the invasive lesions. The combined radiographic–radiomics model (AUC 0.77; 95% CI, 0.69–0.86) performed better than the radiographic model (AUC 0.71; 95% CI, 0.62–0.81) and Rad-score (AUC 0.72; 95% CI, 0.63–0.81) in the validation set. The clinical utility of the individualized prediction nomogram developed using the Rad-score, margin, spiculation, and size was confirmed in the validation set. The decision curve analysis (DCA) indicated that using a model with Rad-score to predict the invasive lesion would be more beneficial than that without Rad-score and the clinical model.

**Conclusions:**

The proposed radiomics-based nomogram that incorporated the Rad-score, margin, spiculation, and size may be utilized as a noninvasive biomarker for the assessment of invasive prediction in patients with pGGNs.

**Key Points:**

*• CT-based radiomics analysis helps invasive prediction manifested as pGGNs.*

*• The combined radiographic–radiomics model may be utilized as a noninvasive biomarker for predicting invasive lesion for pGGNs.*

*• Radiomics-based individual nomogram may serve as a vital decision support tool to identify invasive pGGNs, obviating further workup and blind follow-up.*

**Electronic supplementary material:**

The online version of this article (10.1007/s00330-020-06776-y) contains supplementary material, which is available to authorized users.

## Introduction

The improvement in the computed tomography (CT) scanners and the increasing awareness of the physical examination have led to the detection of the number of ground-glass nodules (GGNs) as well as attracted unprecedented attention. GGN is defined as a nodule with slightly increased density, without obscuring the underlying bronchial structures or vascular margins on high-resolution CT [1]. Based on the presence of solid components, GGNs can be classified into pure GGN (pGGN) and part-solid nodule. Approximately, 20% of lung adenocarcinomas manifested as pGGN on CT imaging and showed stable or extremely slow growth on the follow-up CT as well as favorable prognosis [2–4]. A majority of the international guidelines (including American College of Chest Physicians, National Comprehensive Cancer Network, and British Thoracic Society) have adopted a conservative treatment attitude for pGGNs; yet, in one study [5], 10% of pGGNs ≤ 5 mm showed growth and 1% developed into invasive lesions after 3.5 years. In other two studies [6, 7], up to 52% and 58% of pGGNs progressed during follow-up. These data indicated that many pGGNs were active and should not be neglected. Also, several studies demonstrated that 40.2–55.3% of pGGNs were invasive lesions [8–11]. The appearance of invasive components was considered to be a sign that tumor cells broke the balance of indolent period and entered the rapid growth time. Therefore, it is not appropriate to utilize the same follow-up strategy or intervention for pGGNs with or without invasive components.

Previous studies suggested that large volume, high voxel attenuation, unsmooth margin, pleural indentation, vessel changes, and bubble sign favor the diagnosis of invasive lesions in pGGNs [12–16] that are yet difficult to diagnose based on CT imaging. The reasons are as follows: (1) The measurement and observation of these features are easily affected by several factors (such as reproduction of measurement, scanning parameters, reconstruction algorithm, and observer experience), (2) some features have a low occurrence in pGGNs with limited value for identifying the preinvasive lesions, and (3) most features overlap in invasive and noninvasive lesions.

Artificial intelligence (AI) utilizes radiomics to characterize lung lesions using computer software to extract a large number of predefined high-throughput features, followed by statistical methods to filter the features most relevant to the results. Finally, the method of machine learning is adopted to establish a diagnostic and predictive model [17]. The application of radiomics in the diagnosis and evaluation of pulmonary nodules is currently under intensive focus. Hwang et al [18] investigated the importance of texture in the diagnosis of pGGNs; however, the number of cases was small (64 cases), and hence, fewer features were extracted. Our previous studies [10] have shown that a radiomics-based model has better diagnostic performance for predicting the subcentimeter GGNs, but the present study included both part-solid nodules and pGGNs. Since the solid components on CT imaging exhibit a satisfactory correlation with pathological invasive components [19, 20], the diagnosis of part-solid nodules is uncomplicated, while the diagnosis of pGGNs is difficult and the invasiveness is often underestimated. Therefore, this study aimed to establish a comprehensive model of pGGNs based on the traditional CT features and radiomics to improve the diagnostic accuracy of pGGNs and provide a basis for rational clinical decision-making.

## Materials and methods

### Study population

**Fig. 1:**
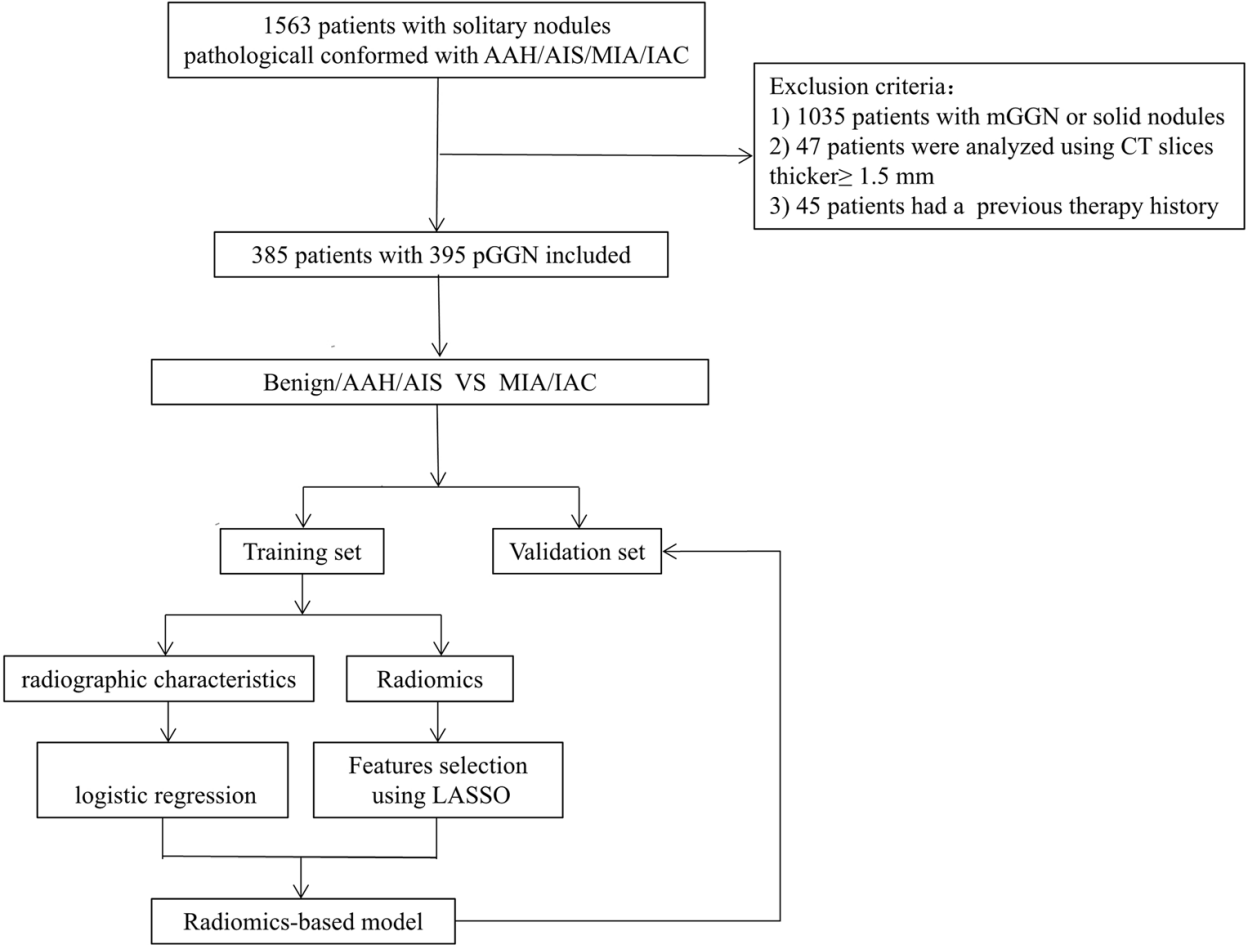
The workflow of the study

This retrospective study was approved by the institutional review board (Grant No. 2017K062), and informed consent was waived. Between January 2011 and October 2017, 1512 consecutive patients underwent complete surgical and were pathologically confirmed with atypical adenomatoid hyperplasia (AAH), adenocarcinoma in situ (AIS), minimal invasive adenocarcinoma (MIA), and invasive pulmonary adenocarcinoma (IPA). The medical records, including clinical characteristics, histopathological results, and serial chest CT scans, were reviewed. Inclusion criteria for the current study were as follows: (1) patients with pure GGN without any solid component on CT imaging, (2) patients with thin-section chest CT (section thickness ≤ 1.5 mm), (3) CT scans that were available and acquired sequentially in one examination within 2 weeks of surgical resection, and (4) patients who underwent complete surgical resection. PGGNs were GGNs without solid components that obscured the lung parenchyma [21] completely, or the underlying lung architecture was viewed with lung window settings (width, 1500 HU; level, − 700 HU). Of these 1512 patients, 1178 were excluded from our study because 1035 had part-solid nodules, 47 were analyzed using CT slices thicker than 1.5 mm, and 45 had a previous therapy history (Fig. 1).

**Fig. 2:**
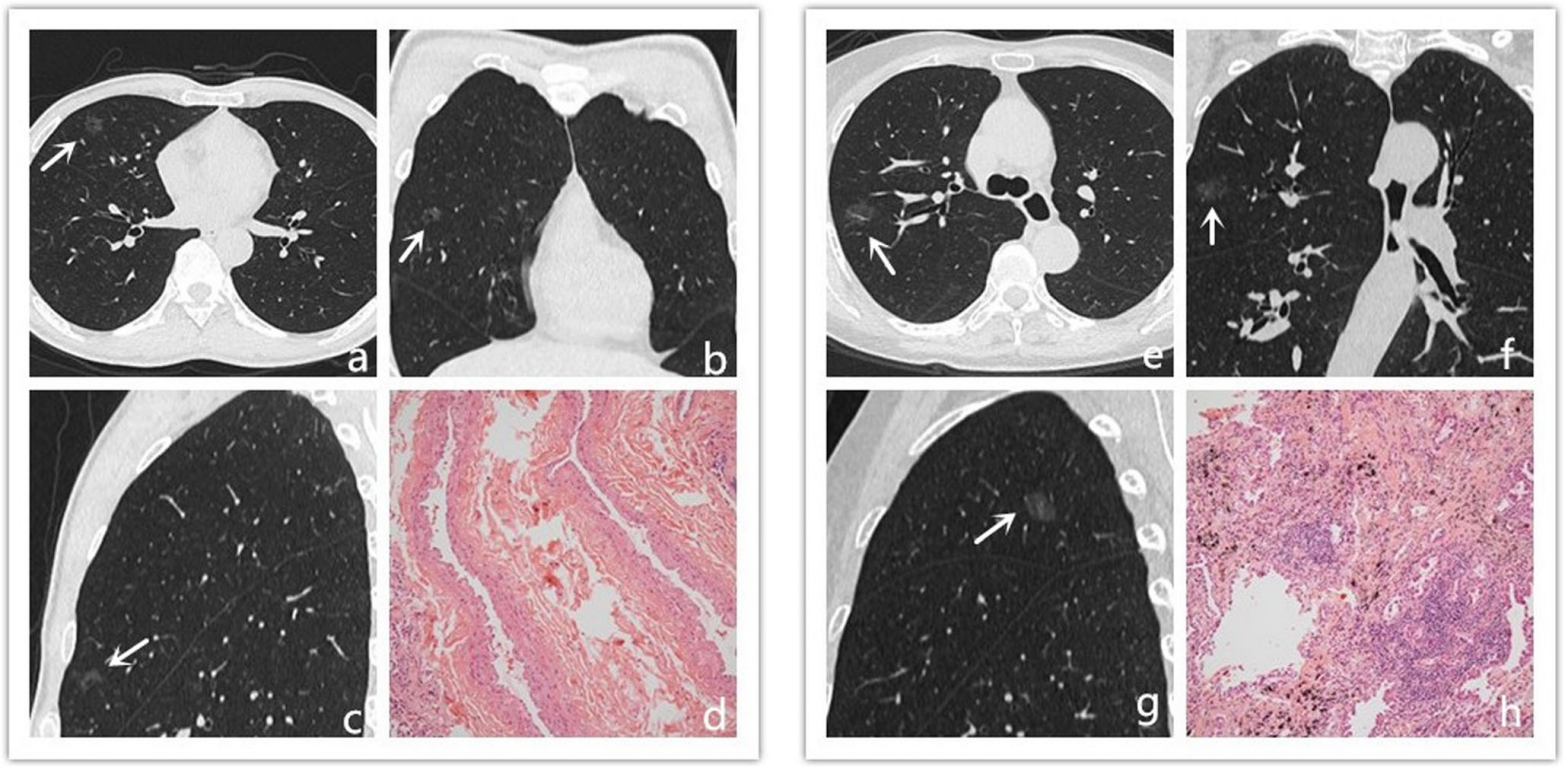
Noninvasive lesion and invasive lesion appearing as pure GGNs. **a**–**d** Transverse, coronal, sagittal, and pathology imaging (hematoxylin and eosin, × 100) of an 11-mm pure GGN in the right middle lobe. This nodule was confirmed as non-neoplastic lesion (fibrosis, with alveolar epithelial hyperplasia and dysplasia, vascular malformations). **e**–**h** Transverse, coronal, sagittal, and pathology imaging (hematoxylin and eosin, × 100) of an 18-mm well-defined pure GGN in the right upper lobe of a 72-year-old woman. This nodule was confirmed as IPA at lobectomy

Finally, 395 pGGNs in 385 patients (121 males, 264 women; mean age, 53.13 ± 12.25 years; range, 23–81 years) fulfilled the criteria and were randomly assigned to a training set (*n* = 277) and a validation set (*n* = 118) (Fig. 2).

We find rare data on the public dataset collected in TCIA (https://www.cancerimagingarchive.net/) for external validation mainly because of the lack of detailed pathological results, and hence used 53 consecutive data from another hospital as the testing set (20 men, 33 women; mean age, 54.04 ± 9.75 years; range, 28–86 years). The diagnostic performance of the model was further verified in the testing set.

### CT scanning

Chest CT imaging was performed using one of the four CT systems: GE Discovery CT750 HD, 64-slice LightSpeed VCT (GE Medical Systems), Somatom Definition Flash, and Somatom Sensation 16 (Siemens Medical Solutions). The detailed scan and reconstruction parameters are listed in Table 1.

**Table 1:** CT scanning parameters

Setting	GE Discovery CT750 HD	LightSpeed VCT	Somatom Definition Flash	Somatom Sensation 16
Tube voltage (kVp)	120	120	120	120
Tube current (mA)	200	200	110	110
Pitch	0.984:f1	0.984:1	1.0	0.8
Collimation	0.625 mm × 64	0.625 mm × 64	0.6 mm × 64	0.75 mm × 16
Rotation time (s/rot)	0.5	0.5	0.33	0.35
SFOV (cm)	50	50	50	50
Slice thickness of reconstruction (mm)	1.25	1.25	1	1
Slice interval of reconstruction (mm)	1.25	1.25	1	1
Reconstruction algorithm	STND	STND	Medium sharp	Medium sharp

### Pathological analysis

All resected specimens were formalin fixed and stained with hematoxylin–eosin in accordance with the routine regulations of our hospital. A board-certified pathologist (10-year experience of pathological diagnosis of lung cancer) reviewed the specimens and recorded the pathological subtype of each tumor according to the International Association for the Study of Lung Cancer (IASLC)/American Thoracic Society (ATS)/European Respiratory Society (ERS). All pGGNs were divided into noninvasive (benign, AAH, and AIS) and invasive (MIA and IPA) groups.

### Evaluation of radiographic characteristics

The radiographic characteristics were assessed independently by two experienced thoracic radiologists (with 6 years and 13 years of experience in chest CT interpretation, respectively), who were blinded to the pathological results. The discrepancies in the interpretation between observers were resolved by consensus. The radiographic characteristics that were analyzed for each pGGN included (1) margin (clear, blurred), (2) lobulation (absent, presence), (3) spiculation (absent, present), (3) bubble lucency (absence, presence), (4) honeycomb sign (absent, present), (5) change in the vessels (absent, present), and (6) pleural attachment including pleural tag and indentation (absent, present). All CT findings were evaluated based on high-resolution CT (HRCT) images.

### Nodule segmentation

An open-source software (3D Slicer, version 4.8; National Institutes of Health; https://www.slicer.org) was used to manually delineate the volume of interest (VOI) of the included 395 pGGNs at the voxel level by one radiologist with 6 years of experience in chest CT interpretation; then, the VOI was confirmed by another radiologist with 13 years of experience in chest CT interpretation. Large vessels and bronchus were excluded from the volume of the nodule. The lung CT Digital Imaging and Communications in Medicine (DICOM) format images were imported into the software for delineation, and then, the images with VOI information were extracted using NII (151 nodules) or NRRD (244 nodules) format for the next step analysis. To assess the segmentation variability, a third radiologist with 3 years of experience in chest CT interpretation independently segmented a random set of 60 nodules.

### Radiomic feature extraction

A total of 396 features, including tumor size, shape, first-order statistics of descriptor values (histogram features), and high-order texture features (gray level co-occurrence matrix and gray level run length), were extracted using Artificial Intelligence Kit software (A.K. software; GE Healthcare). Detailed information about the extracted radiomic features is provided in Appendix E1. Each image was normalized to achieve a nonzero mean and unit variance across the training and validation sets.

### Feature selection, Rad-score building, and diagnostic validation

The interobserver reproducibility evaluation of radiomic feature extraction was performed using intraclass correlation coefficients (ICCs) 0.81–1.00 indicating almost perfect agreement, 0.61–0.80 substantial agreement, and 0.41–0.60 moderate agreement [22]. The features with ICC > 0.80 were input to the least absolute shrinkage and selection operator classifier to establish the radiomic signatures for the distinction between noninvasive and invasive groups. The classifier was trained to determine the optimal parameter using 10-fold cross-validation on the training set.

### Development and clinical utility of individualized prediction nomogram

Multivariate logistic regression analysis using backward stepwise selection was applied to develop the radiographic–radiomics model by incorporating the selected radiomic and radiographic features. The predictive performance of the radiographic model, radiomics model, and the combined model in the training, validation, and testing sets was assessed using the receiver operating characteristic (ROC) curve analysis, and the areas under the curve (AUCs) were established. Then, an individualized prediction nomogram was constructed based on the multivariate logistic regression.

### Statistical analysis

Statistical analysis was conducted using R software (version 3.5.1; http://www.Rproject.org). The statistical significance for all two-sided tests was set at *p* < 0.05.

## Results

### Patients’ profiles

A total of 395 pGGNs were detected in 385 patients in our institution. Of these, one pGGN was detected in 375 patients and two in 10 patients. In addition, 164 pGGNs were pathologically diagnosed as noninvasive lesions (benign nodules, *n* = 52; AAH, *n* = 20; AIS, *n* = 92), while 231 pGGNs were invasive lesions (MIA, *n* = 176; IPA, *n* = 55). One hundred fifteen noninvasive and 162 invasive pure GGNs were grouped in the training set, while 49 noninvasive and 69 invasive pure GGNs in the validation set. In the testing set, 22 pGGNs were pathologically diagnosed as noninvasive lesions (benign nodules, *n* = 5; AAH, *n* = 4; AIS, *n* = 13), while 31 pGGNs were invasive lesions (MIA, *n* = 5; IPA, *n* = 26). Differences in variables among the training, validation, and testing sets were assessed using the independent *t* test or Mann–Whitney *U* test for continuous variables and Fisher’s exact or chi-square test for categorical variables. Any significant differences were not detected in the clinical and radiographic characteristics. The patient profiles for subgroups in the training and validation sets were assimilated based on the invasiveness (Table 2).

**Table 2:** Parameters of patients in noninvasive and invasive groups included in the training set and validation set

Clinical parameters	Data	Noninvasive group (*n* = 164)	Invasive group (*n* = 231)	*p* value
Age (years)	53.13 ± 12.25	53.00 ± 12.25	53.23 ± 12.49	0.841
Maximal tumor diameter (cm)	0.84 ± 0.44	0.74 ± 0.44	0.90 ± 0.44	< 0.001*
Margin				
Clear	346 (87.6)	128 (78.0)	218 (94.4)	< 0.001*
Blurred	49 (12.4)	36 (22.0)	13 (5.6)	
Lobulation				
Absent	200 (50.6)	105 (64.0)	95 (41.1)	< 0.001*
Present	195 (49.4)	59 (36.0)	136 (58.9)	
Spiculation				
Absent	237 (60.0)	127 (77.4)	110 (47.6)	< 0.001*
Present	158 (40.0)	37 (22.6)	121 (52.4)	
Vessel change				
Absent	357 (90.4)	154 (93.9)	203 (87.9)	0.046
Present	38 (9.6)	10 (6.1)	28 (12.1)	
Bubble				
Absent	314 (79.5)	133 (81.1)	181 (78.4)	0.507
Present	82 (20.5)	31 (18.9)	50 (21.6)	
Honeycomb sign				
Absent	380 (96.2)	161 (98.2)	219 (94.8)	0.085
Present	15 (3.8)	3 (1.8)	12 (5.2)	
Pleural attachment				
Absent	331 (83.8)	135 (82.3)	196 (84.8)	0.502
Present	64 (16.2)	29 (17.7)	35 (15.2)	
Location				
RUL	163 (41.3)	68 (41.5)	95 (41.1)	0.086
RML	24 (6.1)	10 (6.1)	14 (6.1)	
RLL	75 (19.0)	31 (18.9)	44 (19.0)	
LUL	88 (22.3)	39 (23.8)	49 (21.2)	
LLL	45 (11.4)	16 (9.8)	29 (12.6)	
Pathological typing				
Benign nodule	52 (13.2)	52 (31.7)		
AAH	20 (5.1)	20 (12.2)		
AIS	92 (23.3)	92 (56.1)		
MIA	176 (44.6)		176 (76.2)	
IPA	55 (13.9)		55 (23.8)	

Ages and size are shown as mean ± standard deviation; other data are the number of patients with the percentage in parentheses. *P* value is derived from the univariable association analyses between clinical parameter and invasiveness of pGGNs

*RUL* right upper lobe, *RML* right middle lobe, *RLL* right lower lobe, *LUL* left upper lobe, *LLL* left lower lobe, *AAH* atypical adenomatoid hyperplasia, *AIS* adenocarcinoma in situ, *MIA* minimal invasive adenocarcinoma, *IPA* invasive pulmonary adenocarcinoma

*p value < 0.05

**Fig. 3:**
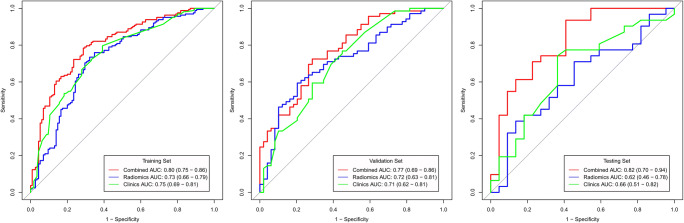
The AUC of Rad-score, radiographic model, and combined model in the training set, validation set, and testing set. The predictive performance of the combined model for an invasive lesion of pGGNs was better than that of the radiographic model and Rad-score in the training, validation, and testing sets

Furthermore, statistically significant differences were observed between the noninvasive and invasive groups with respect to size, margin, spiculation, and lobulation (*p* < 0.001). Multivariate analysis revealed significant differences in the size, margin, and spiculation (*p* = 0.001, *p* < 0.001, and *p* < 0.001, respectively). Subsequently, all three parameters were selected to establish the radiographic model. The AUC of the radiographic model was 0.75 (95% CI, 0.69–0.81) in the training set, 0.71 (95% CI, 0.62–0.81) in the validation set, and 0.66 (95% CI, 0.51–0.82) in the testing set (Fig. 3).

### Feature selection, Rad-score building, and diagnostic validation

**Fig. 4:**
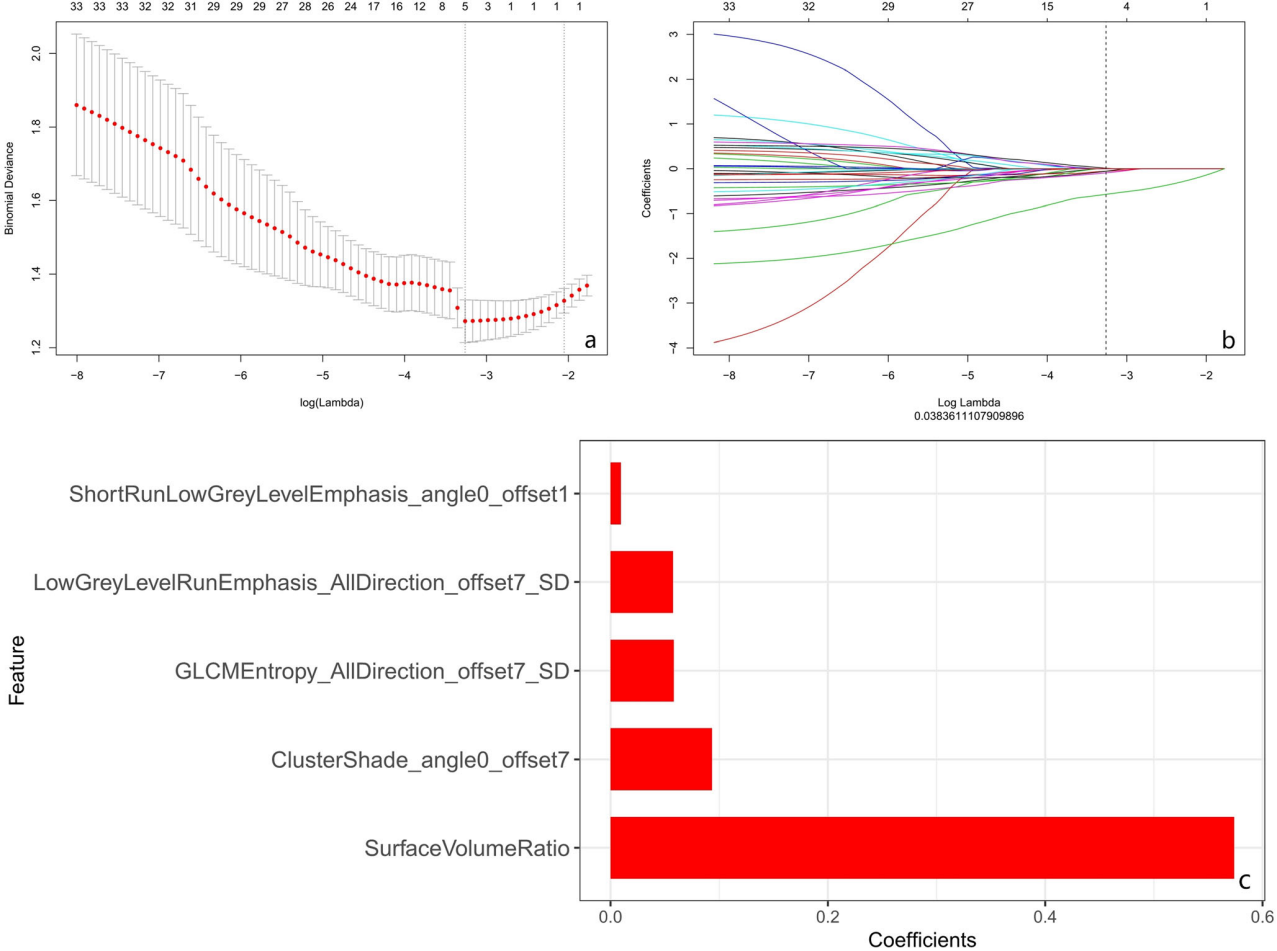
Texture feature selection using the least absolute shrinkage and the histogram of the Rad-score based on the selected features. **a** Selection of the tuning parameter (*λ*) in the LASSO model via 10-fold cross-validation based on minimum criteria. Binomial deviances from the LASSO regression cross-validation procedure were plotted as a function of log (*λ*). The optimal *λ* value of 0.038 was selected. **b** The black vertical line was drawn at the value selected using 10-fold cross-validation in **a**. The 5 resulting features with nonzero coefficients were indicated in the plot. **c** The *y*-axis indicates the selected five radiomics, and the *x*-axis represents the coefficient of radiomics

Five features with nonzero coefficients were selected to establish the Rad-score using a least absolute shrinkage and selection operator (LASSO) logistic regression model (*λ* = 0.038) (Fig. 4a, b) after assessing the reproducibility based on the resegmentation data.

Rad-score = − 0.093 × ClusterShade_angle0_offset7 − 0.058 × GLCMEntropy_AllDirection_offset7_SD − 0.057 × LowGreyLevelRunEmphasis_AllDirection_offset7_SD + 0.010 × ShortRunLowGreyLevelEmphasis_angle0_offset1 − 0.574 × SurfaceVolumeRatio + 0.368

The histogram of the Rad-score is presented in Fig. 4 c. The detail information of the two sets is illustrated in Appendix E2.

A significant difference was detected in the Rad-score between noninvasive and invasive groups in the training set (0.10 ± 0.60 vs. 0.56 ± 0.51; *p* < 0.001), which was then confirmed in the validation set (− 0.03 ± 0.71 vs. 0.47 ± 0.60, *p* < 0.001). The Rad-score predicting the invasive pGGNs yielded a C-index of 0.73 (95% CI, 0.66–0.79) in the training set, 0.72 (95% CI, 0.63–0.81) in the validation set, and 0.62 (95% CI, 0.46–0.78) in the testing set. The AUC of the combined radiographic–radiomics model was 0.80 (95% CI, 0.75–0.86) in the training set, 0.77 (95% CI, 0.69–0.86) in the validation set, and 0.82 (95% CI, 0.70–0.94) in the testing set (Fig. 3).

Although the difference in tumor size between the noninvasive and invasive groups was statistically significant (0.74 cm vs. 0.90 cm, *p* < 0.001), the AUC of size for classifying noninvasive and invasive lesions was only 0.68 in the training set and 0.65 in the validation set. The surface–volume ratio is the strongest radiomic feature with a coefficient of 0.574 in the Rad-score formula. However, the AUC of surface–volume ratio for classifying noninvasive and invasive lesions was 0.72 in the training set and 0.70 in the validation set. The AUC of size and surface–volume ratio for classifying noninvasive and invasive lesions were all lower than those of the radiographic model (0.75 and 0.71, respectively), Rad scores (0.73 and 0.72, respectively), and the combined model (0.80 and 0.77, respectively). Furthermore, we had performed DeLong’s test to analyze the difference between the AUC of size, surface–volume ratio, and the combined model in the validation set. The result showed the difference was not significant (*p* = 0.38, *p* = 0.12), which may be affected by the amount of data and needed further verification.

### Construction of individualized prediction nomogram and clinical utility

**Fig. 5:**
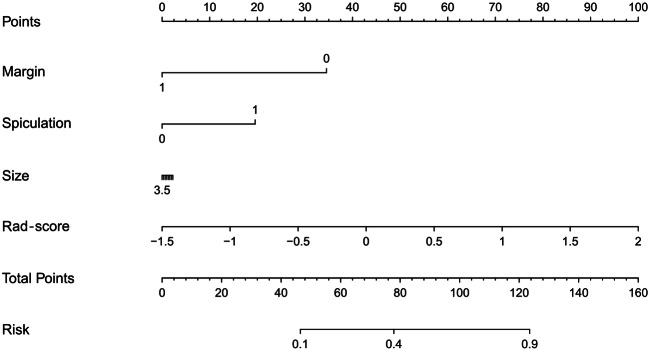
Radiomics-based nomogram was developed in the training set, and the Rad-score, margin, spiculation, and size were incorporated

A logistic regression analysis using backward stepwise selection identified the Rad-score, margin, spiculation, and size as independent predictors, which were incorporated to develop an individualized prediction nomogram (Fig. 5).

**Fig. 6:**
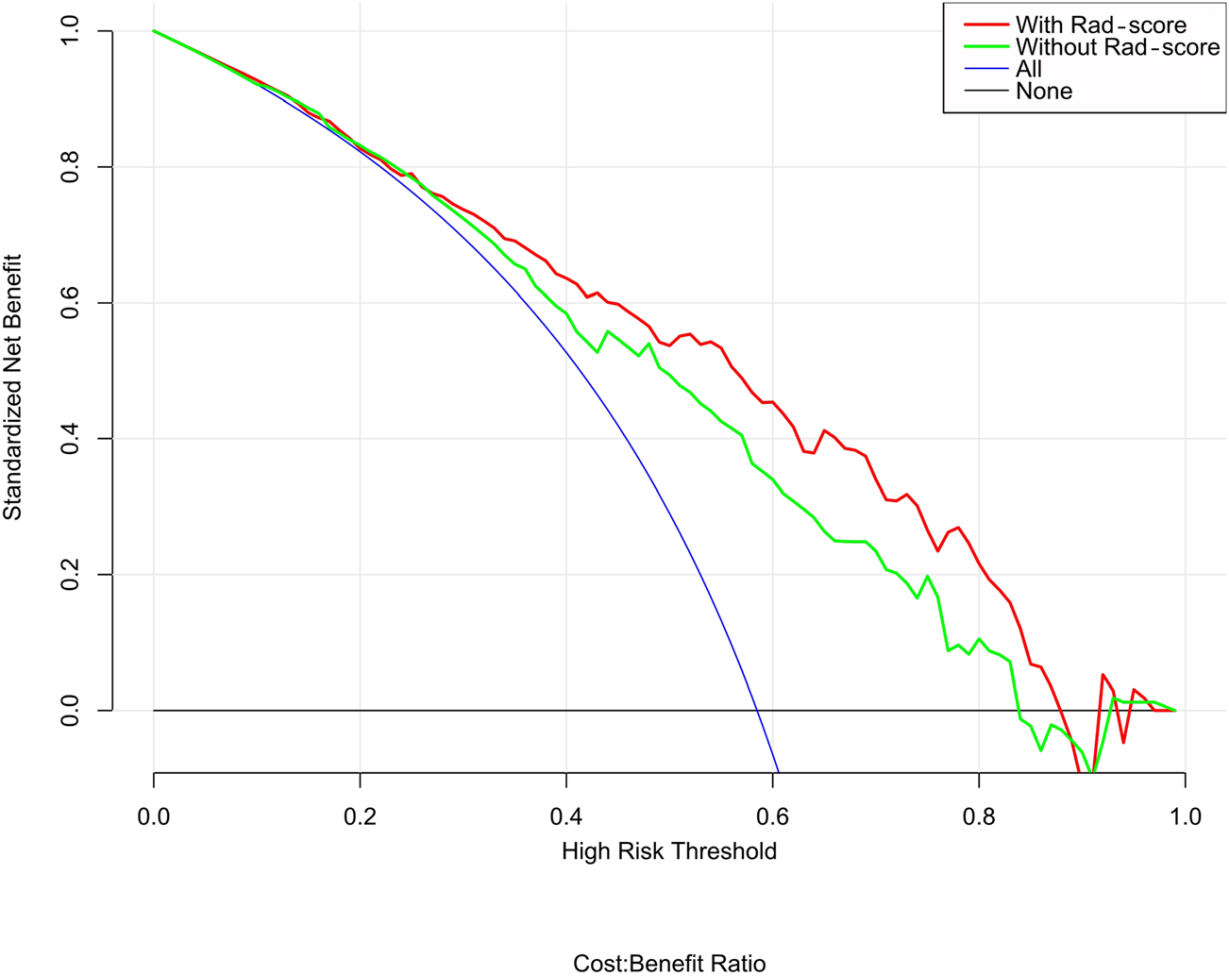
Decision curve analysis for the model with and without Rad-score. The decision curve showed that if the threshold probability of a patient or a doctor is > 10%, using a model with the Rad-score to predict the invasive lesion would be more beneficial than that without the Rad-score

The decision curve analysis (DCA) for the individualized prediction nomogram is presented in Fig. 6. The decision curve showed that if the threshold probability of a patient or doctor was > 10%, using a model with Rad-score to predict the invasive lesion would be more beneficial than that without the Rad-score and the clinical model.

## Discussion

The present study aimed to assess the value of radiomics in the diagnosis of pGGNs. We systematically evaluated the traditional radiographic features of lung cancer and extracted more than 400 radiomics. Then, a combined invasiveness-predicting model incorporated with Rad-score, spiculation, and margin was developed and validated using backward stepwise selection. Finally, a convenient nomogram incorporated with Rad-score, spiculation, margin, and size was established.

Several studies have analyzed and compared the value of traditional CT features and radiomics in the diagnosis of GGN. Fan et al [23] showed that the imaging radiomics–based model (AUC = 0.936) is superior to traditional CT feature–based model (AUC = 0.857) in identifying invasive lesions, and only the radiomics model is considered to be an independent predictor of invasive GGNs. In the case of pGGNs, previous studies were mainly based on traditional CT features [12, 13, 24]. The current study, for the first time, combined the traditional CT features and radiomics to establish an individual predictive model of pGGNs. Liu et al [25] also confirmed the advantage of radiomics in the diagnosis of invasive GGN and showed that the performance of combined radiomics model was better than that of the traditional radiographic features. Xue et al [26] showed that despite the lobulated border and the presence of pleural effectuated a significant difference between preinvasive and invasive lesions, they were not included in the final predictive nomogram. Although many studies have assessed traditional CT features for invasive predicting, they could not result in a systematic and consistent criterion to integrate the contribution of different risk factors to achieve a probabilistic forecast of MIA/IPA in GGN because the CT features of early-stage lung cancer are often atypical, and the assessment of these signs requires extensive diagnostic experience. The current result showed that Rad-score (AUC = 0.72) and radiographic model (AUC = 0.71) had similar diagnostic efficiency; however, both were lower than that of the combined model (AUC = 0.77). The result of radiographic feature in this study was confirmed by an expert experienced with the diagnosis of small pulmonary nodules, which improved the accuracy of the radiographic features. Thus, the similarity in the performance of the radiographic model and Rad-score could be detected.

The size of the nodules is a vital parameter for assessing the invasiveness of GGNs. Previous studies showed that the cutoff value of 10 mm is an optimal predictor for invasive lesions in pGGNs with 100% specificity [27]. Another study demonstrated that the diameter > 16.4 mm was favored in invasive adenocarcinoma for pGGNs > 10 mm [28]. However, Wu et al [8] showed that the size of the nodule could not differentiate the invasive lesions from preinvasive lesions, and the average size of the nodules in the study was < 10 mm. In the current study, multivariate analysis revealed that the size was not an independent predictor. However, the final nomogram included the parameter of size and the performance was not optimal. Similar to the data of Wu et al [8], the average size of the pGGNs in the current study was < 10 mm, which might be the differential factor. Thus, the performance of size as a predictor of invasive lesion correlated with nodule size.

Nevertheless, the present study had several limitations. First, it was a single-center retrospective study, and only surgically resected pGGNs were included; thus, the verification bias was inherent to our study design. Second, a reliable and robust automatic method is essential to simplify the complex and time-consuming boundary extraction process. Third, the result showed similar performance of the radiographic model and Rad-score with respect to the prediction invasiveness of pGGNs; however, we did not conduct further analysis and could not assess the correlation between radiomics and radiographic features.

In conclusion, radiomic features provide a crucial reference for the prediction of invasiveness. These accidental pGGNs would receive a timely and reasonable solution, avoiding the blind follow-up and radical invasive treatment. Therefore, with the gradual development of artificial intelligence technology, this quantitative nomogram prediction model based on the radiomic features of CT imaging, used for the differential noninvasive lesion and invasive lesion of pGGNs, may have broad clinical applications.

## Electronic supplementary material

ESM 1 (DOCX 76 kb)

## Abbreviations

AAH: Atypical adenomatoid hyperplasia
AIS: Adenocarcinoma in situ
AUC: Area under the curve
DCA: Decision curve analysis
DICOM: Digital Imaging and Communications in Medicine
GGNs: Ground-glass nodules
IPA: Invasive pulmonary adenocarcinoma
MIA: Minimal invasive adenocarcinoma
pGGN: Pure ground-glass nodule
ROC: Receiver operating characteristic
VOI: Volume of interest
