# Supplementary material for: Radiomics for lung adenocarcinoma manifesting as pure ground-glass nodules: invasive prediction
Source: Eur Radiol. 2020 Mar 11;30(7):3650–9. doi: 10.1007/s00330-020-06776-y (PMC7305264; doi:10.1007/s00330-020-06776-y)
Supplement: Supplementary file 1 — (DOCX 76 kb) [file 330_2020_6776_MOESM1_ESM.docx]

**Supplementary Material**

**Appendix E1**

| FrequencySize |
| --- |
| MaxIntensity |
| MeanDeviation |
| MeanValue |
| MedianIntensity |
| MinIntensity |
| Percentile10 |
| Percentile15 |
| Percentile20 |
| Percentile25 |
| Percentile30 |
| Percentile35 |
| Percentile40 |
| Percentile45 |
| Percentile5 |
| Percentile50 |
| Percentile55 |
| Percentile60 |
| Percentile65 |
| Percentile70 |
| Percentile75 |
| Percentile80 |
| Percentile85 |
| Percentile90 |
| Percentile95 |
| Quantile0.025 |
| Quantile0.25 |
| Quantile0.5 |
| Quantile0.75 |
| Quantile0.975 |
| RMS |
| Range |
| RelativeDeviation |
| Variance |
| VolumeCount |
| VoxelValueSum |
| histogramEnergy |
| histogramEntropy |
| kurtosis |
| skewness |
| stdDeviation |
| uniformity |
| ClusterProminence_AllDirection_offset1 |
| ClusterProminence_AllDirection_offset1_SD |
| ClusterProminence_AllDirection_offset4 |
| ClusterProminence_AllDirection_offset4_SD |
| ClusterProminence_AllDirection_offset7 |
| ClusterProminence_AllDirection_offset7_SD |
| ClusterProminence_angle0_offset1 |
| ClusterProminence_angle0_offset4 |
| ClusterProminence_angle0_offset7 |
| ClusterProminence_angle135_offset1 |
| ClusterProminence_angle135_offset4 |
| ClusterProminence_angle135_offset7 |
| ClusterProminence_angle45_offset1 |
| ClusterProminence_angle45_offset4 |
| ClusterProminence_angle45_offset7 |
| ClusterProminence_angle90_offset1 |
| ClusterProminence_angle90_offset4 |
| ClusterProminence_angle90_offset7 |
| ClusterShade_AllDirection_offset1 |
| ClusterShade_AllDirection_offset1_SD |
| ClusterShade_AllDirection_offset4 |
| ClusterShade_AllDirection_offset4_SD |
| ClusterShade_AllDirection_offset7 |
| ClusterShade_AllDirection_offset7_SD |
| ClusterShade_angle0_offset1 |
| ClusterShade_angle0_offset4 |
| ClusterShade_angle0_offset7 |
| ClusterShade_angle135_offset1 |
| ClusterShade_angle135_offset4 |
| ClusterShade_angle135_offset7 |
| ClusterShade_angle45_offset1 |
| ClusterShade_angle45_offset4 |
| ClusterShade_angle45_offset7 |
| ClusterShade_angle90_offset1 |
| ClusterShade_angle90_offset4 |
| ClusterShade_angle90_offset7 |
| Correlation_AllDirection_offset1 |
| Correlation_AllDirection_offset1_SD |
| Correlation_AllDirection_offset4 |
| Correlation_AllDirection_offset4_SD |
| Correlation_AllDirection_offset7 |
| Correlation_AllDirection_offset7_SD |
| Correlation_angle0_offset1 |
| Correlation_angle0_offset4 |
| Correlation_angle0_offset7 |
| Correlation_angle135_offset1 |
| Correlation_angle135_offset4 |
| Correlation_angle135_offset7 |
| Correlation_angle45_offset1 |
| Correlation_angle45_offset4 |
| Correlation_angle45_offset7 |
| Correlation_angle90_offset1 |
| Correlation_angle90_offset4 |
| Correlation_angle90_offset7 |
| GLCMEnergy_AllDirection_offset1 |
| GLCMEnergy_AllDirection_offset1_SD |
| GLCMEnergy_AllDirection_offset4 |
| GLCMEnergy_AllDirection_offset4_SD |
| GLCMEnergy_AllDirection_offset7 |
| GLCMEnergy_AllDirection_offset7_SD |
| GLCMEnergy_angle0_offset1 |
| GLCMEnergy_angle0_offset4 |
| GLCMEnergy_angle0_offset7 |
| GLCMEnergy_angle135_offset1 |
| GLCMEnergy_angle135_offset4 |
| GLCMEnergy_angle135_offset7 |
| GLCMEnergy_angle45_offset1 |
| GLCMEnergy_angle45_offset4 |
| GLCMEnergy_angle45_offset7 |
| GLCMEnergy_angle90_offset1 |
| GLCMEnergy_angle90_offset4 |
| GLCMEnergy_angle90_offset7 |
| GLCMEntropy_AllDirection_offset1 |
| GLCMEntropy_AllDirection_offset1_SD |
| GLCMEntropy_AllDirection_offset4 |
| GLCMEntropy_AllDirection_offset4_SD |
| GLCMEntropy_AllDirection_offset7 |
| GLCMEntropy_AllDirection_offset7_SD |
| GLCMEntropy_angle0_offset1 |
| GLCMEntropy_angle0_offset4 |
| GLCMEntropy_angle0_offset7 |
| GLCMEntropy_angle135_offset1 |
| GLCMEntropy_angle135_offset4 |
| GLCMEntropy_angle135_offset7 |
| GLCMEntropy_angle45_offset1 |
| GLCMEntropy_angle45_offset4 |
| GLCMEntropy_angle45_offset7 |
| GLCMEntropy_angle90_offset1 |
| GLCMEntropy_angle90_offset4 |
| GLCMEntropy_angle90_offset7 |
| HaralickCorrelation_AllDirection_offset1 |
| HaralickCorrelation_AllDirection_offset1_SD |
| HaralickCorrelation_AllDirection_offset4 |
| HaralickCorrelation_AllDirection_offset4_SD |
| HaralickCorrelation_AllDirection_offset7 |
| HaralickCorrelation_AllDirection_offset7_SD |
| HaralickCorrelation_angle0_offset1 |
| HaralickCorrelation_angle0_offset4 |
| HaralickCorrelation_angle0_offset7 |
| HaralickCorrelation_angle135_offset1 |
| HaralickCorrelation_angle135_offset4 |
| HaralickCorrelation_angle135_offset7 |
| HaralickCorrelation_angle45_offset1 |
| HaralickCorrelation_angle45_offset4 |
| HaralickCorrelation_angle45_offset7 |
| HaralickCorrelation_angle90_offset1 |
| HaralickCorrelation_angle90_offset4 |
| HaralickCorrelation_angle90_offset7 |
| Inertia_AllDirection_offset1 |
| Inertia_AllDirection_offset1_SD |
| Inertia_AllDirection_offset4 |
| Inertia_AllDirection_offset4_SD |
| Inertia_AllDirection_offset7 |
| Inertia_AllDirection_offset7_SD |
| Inertia_angle0_offset1 |
| Inertia_angle0_offset4 |
| Inertia_angle0_offset7 |
| Inertia_angle135_offset1 |
| Inertia_angle135_offset4 |
| Inertia_angle135_offset7 |
| Inertia_angle45_offset1 |
| Inertia_angle45_offset4 |
| Inertia_angle45_offset7 |
| Inertia_angle90_offset1 |
| Inertia_angle90_offset4 |
| Inertia_angle90_offset7 |
| InverseDifferenceMoment_AllDirection_offset1 |
| InverseDifferenceMoment_AllDirection_offset1_SD |
| InverseDifferenceMoment_AllDirection_offset4 |
| InverseDifferenceMoment_AllDirection_offset4_SD |
| InverseDifferenceMoment_AllDirection_offset7 |
| InverseDifferenceMoment_AllDirection_offset7_SD |
| InverseDifferenceMoment_angle0_offset1 |
| InverseDifferenceMoment_angle0_offset4 |
| InverseDifferenceMoment_angle0_offset7 |
| InverseDifferenceMoment_angle135_offset1 |
| InverseDifferenceMoment_angle135_offset4 |
| InverseDifferenceMoment_angle135_offset7 |
| InverseDifferenceMoment_angle45_offset1 |
| InverseDifferenceMoment_angle45_offset4 |
| InverseDifferenceMoment_angle45_offset7 |
| InverseDifferenceMoment_angle90_offset1 |
| InverseDifferenceMoment_angle90_offset4 |
| InverseDifferenceMoment_angle90_offset7 |
| AngularSecondMoment |
| HaraEntroy |
| HaraVariance |
| contrast |
| differenceEntropy |
| differenceVariance |
| inverseDifferenceMoment |
| sumAverage |
| sumEntropy |
| sumVariance |
| GreyLevelNonuniformity_AllDirection_offset1 |
| GreyLevelNonuniformity_AllDirection_offset1_SD |
| GreyLevelNonuniformity_AllDirection_offset4 |
| GreyLevelNonuniformity_AllDirection_offset4_SD |
| GreyLevelNonuniformity_AllDirection_offset7 |
| GreyLevelNonuniformity_AllDirection_offset7_SD |
| GreyLevelNonuniformity_angle0_offset1 |
| GreyLevelNonuniformity_angle0_offset4 |
| GreyLevelNonuniformity_angle0_offset7 |
| GreyLevelNonuniformity_angle135_offset1 |
| GreyLevelNonuniformity_angle135_offset4 |
| GreyLevelNonuniformity_angle135_offset7 |
| GreyLevelNonuniformity_angle45_offset1 |
| GreyLevelNonuniformity_angle45_offset4 |
| GreyLevelNonuniformity_angle45_offset7 |
| GreyLevelNonuniformity_angle90_offset1 |
| GreyLevelNonuniformity_angle90_offset4 |
| GreyLevelNonuniformity_angle90_offset7 |
| HighGreyLevelRunEmphasis_AllDirection_offset1 |
| HighGreyLevelRunEmphasis_AllDirection_offset1_SD |
| HighGreyLevelRunEmphasis_AllDirection_offset4 |
| HighGreyLevelRunEmphasis_AllDirection_offset4_SD |
| HighGreyLevelRunEmphasis_AllDirection_offset7 |
| HighGreyLevelRunEmphasis_AllDirection_offset7_SD |
| HighGreyLevelRunEmphasis_angle0_offset1 |
| HighGreyLevelRunEmphasis_angle0_offset4 |
| HighGreyLevelRunEmphasis_angle0_offset7 |
| HighGreyLevelRunEmphasis_angle135_offset1 |
| HighGreyLevelRunEmphasis_angle135_offset4 |
| HighGreyLevelRunEmphasis_angle135_offset7 |
| HighGreyLevelRunEmphasis_angle45_offset1 |
| HighGreyLevelRunEmphasis_angle45_offset4 |
| HighGreyLevelRunEmphasis_angle45_offset7 |
| HighGreyLevelRunEmphasis_angle90_offset1 |
| HighGreyLevelRunEmphasis_angle90_offset4 |
| HighGreyLevelRunEmphasis_angle90_offset7 |
| LongRunEmphasis_AllDirection_offset1 |
| LongRunEmphasis_AllDirection_offset1_SD |
| LongRunEmphasis_AllDirection_offset4 |
| LongRunEmphasis_AllDirection_offset4_SD |
| LongRunEmphasis_AllDirection_offset7 |
| LongRunEmphasis_AllDirection_offset7_SD |
| LongRunEmphasis_angle0_offset1 |
| LongRunEmphasis_angle0_offset4 |
| LongRunEmphasis_angle0_offset7 |
| LongRunEmphasis_angle135_offset1 |
| LongRunEmphasis_angle135_offset4 |
| LongRunEmphasis_angle135_offset7 |
| LongRunEmphasis_angle45_offset1 |
| LongRunEmphasis_angle45_offset4 |
| LongRunEmphasis_angle45_offset7 |
| LongRunEmphasis_angle90_offset1 |
| LongRunEmphasis_angle90_offset4 |
| LongRunEmphasis_angle90_offset7 |
| LongRunHighGreyLevelEmphasis_AllDirection_offset1 |
| LongRunHighGreyLevelEmphasis_AllDirection_offset1_SD |
| LongRunHighGreyLevelEmphasis_AllDirection_offset4 |
| LongRunHighGreyLevelEmphasis_AllDirection_offset4_SD |
| LongRunHighGreyLevelEmphasis_AllDirection_offset7 |
| LongRunHighGreyLevelEmphasis_AllDirection_offset7_SD |
| LongRunHighGreyLevelEmphasis_angle0_offset1 |
| LongRunHighGreyLevelEmphasis_angle0_offset4 |
| LongRunHighGreyLevelEmphasis_angle0_offset7 |
| LongRunHighGreyLevelEmphasis_angle135_offset1 |
| LongRunHighGreyLevelEmphasis_angle135_offset4 |
| LongRunHighGreyLevelEmphasis_angle135_offset7 |
| LongRunHighGreyLevelEmphasis_angle45_offset1 |
| LongRunHighGreyLevelEmphasis_angle45_offset4 |
| LongRunHighGreyLevelEmphasis_angle45_offset7 |
| LongRunHighGreyLevelEmphasis_angle90_offset1 |
| LongRunHighGreyLevelEmphasis_angle90_offset4 |
| LongRunHighGreyLevelEmphasis_angle90_offset7 |
| LongRunLowGreyLevelEmphasis_AllDirection_offset1 |
| LongRunLowGreyLevelEmphasis_AllDirection_offset1_SD |
| LongRunLowGreyLevelEmphasis_AllDirection_offset4 |
| LongRunLowGreyLevelEmphasis_AllDirection_offset4_SD |
| LongRunLowGreyLevelEmphasis_AllDirection_offset7 |
| LongRunLowGreyLevelEmphasis_AllDirection_offset7_SD |
| LongRunLowGreyLevelEmphasis_angle0_offset1 |
| LongRunLowGreyLevelEmphasis_angle0_offset4 |
| LongRunLowGreyLevelEmphasis_angle0_offset7 |
| LongRunLowGreyLevelEmphasis_angle135_offset1 |
| LongRunLowGreyLevelEmphasis_angle135_offset4 |
| LongRunLowGreyLevelEmphasis_angle135_offset7 |
| LongRunLowGreyLevelEmphasis_angle45_offset1 |
| LongRunLowGreyLevelEmphasis_angle45_offset4 |
| LongRunLowGreyLevelEmphasis_angle45_offset7 |
| LongRunLowGreyLevelEmphasis_angle90_offset1 |
| LongRunLowGreyLevelEmphasis_angle90_offset4 |
| LongRunLowGreyLevelEmphasis_angle90_offset7 |
| LowGreyLevelRunEmphasis_AllDirection_offset1 |
| LowGreyLevelRunEmphasis_AllDirection_offset1_SD |
| LowGreyLevelRunEmphasis_AllDirection_offset4 |
| LowGreyLevelRunEmphasis_AllDirection_offset4_SD |
| LowGreyLevelRunEmphasis_AllDirection_offset7 |
| LowGreyLevelRunEmphasis_AllDirection_offset7_SD |
| LowGreyLevelRunEmphasis_angle0_offset1 |
| LowGreyLevelRunEmphasis_angle0_offset4 |
| LowGreyLevelRunEmphasis_angle0_offset7 |
| LowGreyLevelRunEmphasis_angle135_offset1 |
| LowGreyLevelRunEmphasis_angle135_offset4 |
| LowGreyLevelRunEmphasis_angle135_offset7 |
| LowGreyLevelRunEmphasis_angle45_offset1 |
| LowGreyLevelRunEmphasis_angle45_offset4 |
| LowGreyLevelRunEmphasis_angle45_offset7 |
| LowGreyLevelRunEmphasis_angle90_offset1 |
| LowGreyLevelRunEmphasis_angle90_offset4 |
| LowGreyLevelRunEmphasis_angle90_offset7 |
| RunLengthNonuniformity_AllDirection_offset1 |
| RunLengthNonuniformity_AllDirection_offset1_SD |
| RunLengthNonuniformity_AllDirection_offset4 |
| RunLengthNonuniformity_AllDirection_offset4_SD |
| RunLengthNonuniformity_AllDirection_offset7 |
| RunLengthNonuniformity_AllDirection_offset7_SD |
| RunLengthNonuniformity_angle0_offset1 |
| RunLengthNonuniformity_angle0_offset4 |
| RunLengthNonuniformity_angle0_offset7 |
| RunLengthNonuniformity_angle135_offset1 |
| RunLengthNonuniformity_angle135_offset4 |
| RunLengthNonuniformity_angle135_offset7 |
| RunLengthNonuniformity_angle45_offset1 |
| RunLengthNonuniformity_angle45_offset4 |
| RunLengthNonuniformity_angle45_offset7 |
| RunLengthNonuniformity_angle90_offset1 |
| RunLengthNonuniformity_angle90_offset4 |
| RunLengthNonuniformity_angle90_offset7 |
| ShortRunEmphasis_AllDirection_offset1 |
| ShortRunEmphasis_AllDirection_offset1_SD |
| ShortRunEmphasis_AllDirection_offset4 |
| ShortRunEmphasis_AllDirection_offset4_SD |
| ShortRunEmphasis_AllDirection_offset7 |
| ShortRunEmphasis_AllDirection_offset7_SD |
| ShortRunEmphasis_angle0_offset1 |
| ShortRunEmphasis_angle0_offset4 |
| ShortRunEmphasis_angle0_offset7 |
| ShortRunEmphasis_angle135_offset1 |
| ShortRunEmphasis_angle135_offset4 |
| ShortRunEmphasis_angle135_offset7 |
| ShortRunEmphasis_angle45_offset1 |
| ShortRunEmphasis_angle45_offset4 |
| ShortRunEmphasis_angle45_offset7 |
| ShortRunEmphasis_angle90_offset1 |
| ShortRunEmphasis_angle90_offset4 |
| ShortRunEmphasis_angle90_offset7 |
| ShortRunHighGreyLevelEmphasis_AllDirection_offset1 |
| ShortRunHighGreyLevelEmphasis_AllDirection_offset1_SD |
| ShortRunHighGreyLevelEmphasis_AllDirection_offset4 |
| ShortRunHighGreyLevelEmphasis_AllDirection_offset4_SD |
| ShortRunHighGreyLevelEmphasis_AllDirection_offset7 |
| ShortRunHighGreyLevelEmphasis_AllDirection_offset7_SD |
| ShortRunHighGreyLevelEmphasis_angle0_offset1 |
| ShortRunHighGreyLevelEmphasis_angle0_offset4 |
| ShortRunHighGreyLevelEmphasis_angle0_offset7 |
| ShortRunHighGreyLevelEmphasis_angle135_offset1 |
| ShortRunHighGreyLevelEmphasis_angle135_offset4 |
| ShortRunHighGreyLevelEmphasis_angle135_offset7 |
| ShortRunHighGreyLevelEmphasis_angle45_offset1 |
| ShortRunHighGreyLevelEmphasis_angle45_offset4 |
| ShortRunHighGreyLevelEmphasis_angle45_offset7 |
| ShortRunHighGreyLevelEmphasis_angle90_offset1 |
| ShortRunHighGreyLevelEmphasis_angle90_offset4 |
| ShortRunHighGreyLevelEmphasis_angle90_offset7 |
| ShortRunLowGreyLevelEmphasis_AllDirection_offset1 |
| ShortRunLowGreyLevelEmphasis_AllDirection_offset1_SD |
| ShortRunLowGreyLevelEmphasis_AllDirection_offset4 |
| ShortRunLowGreyLevelEmphasis_AllDirection_offset4_SD |
| ShortRunLowGreyLevelEmphasis_AllDirection_offset7 |
| ShortRunLowGreyLevelEmphasis_AllDirection_offset7_SD |
| ShortRunLowGreyLevelEmphasis_angle0_offset1 |
| ShortRunLowGreyLevelEmphasis_angle0_offset4 |
| ShortRunLowGreyLevelEmphasis_angle0_offset7 |
| ShortRunLowGreyLevelEmphasis_angle135_offset1 |
| ShortRunLowGreyLevelEmphasis_angle135_offset4 |
| ShortRunLowGreyLevelEmphasis_angle135_offset7 |
| ShortRunLowGreyLevelEmphasis_angle45_offset1 |
| ShortRunLowGreyLevelEmphasis_angle45_offset4 |
| ShortRunLowGreyLevelEmphasis_angle45_offset7 |
| ShortRunLowGreyLevelEmphasis_angle90_offset1 |
| ShortRunLowGreyLevelEmphasis_angle90_offset4 |
| ShortRunLowGreyLevelEmphasis_angle90_offset7 |
| Compactness1 |
| Compactness2 |
| Maximum3DDiameter |
| SphericalDisproportion |
| Sphericity |
| SurfaceArea |
| SurfaceVolumeRatio |
| VolumeCC |
| VolumeMM |
| SizeZoneVariability |
| HighIntensityEmphasis |
| HighIntensityLargeAreaEmphasis |
| HighIntensitySmallAreaEmphasis |
| IntensityVariability |
| LargeAreaEmphasis |
| LowIntensityEmphasis |
| LowIntensityLargeAreaEmphasis |
| LowIntensitySmallAreaEmphasis |
| SmallAreaEmphasis |
| ZonePercentage |

**Appendix E2**

**Radscore_training**

| Label | ClusterShade_angle0_offset7 | GLCMEntropy_AllDirection_offset7_SD | LowGreyLevelRunEmphasis_AllDirection_offset7_SD | ShortRunLowGreyLevelEmphasis_angle0_offset1 | SurfaceVolumeRatio | radscore |
| --- | --- | --- | --- | --- | --- | --- |
| 0 | -0,15086 | -0,51041 | -0,1289 | -0,63867 | 2,858592 | -1,22801 |
| 0 | 2,998451 | -0,56207 | -0,1289 | -0,93835 | -1,57194 | 1,021006 |
| 0 | -0,15086 | -0,58662 | -0,1289 | 0,095607 | -1,46625 | 1,26575 |
| 0 | -0,14852 | -0,34435 | 6,83855 | 1,353297 | -0,97743 | 0,582304 |
| 0 | -0,21732 | 0,210251 | -0,1289 | -0,85915 | -0,04664 | 0,401638 |
| 0 | 0,281277 | 0,817972 | -0,1289 | -0,93219 | 0,005928 | 0,288818 |
| 1 | -0,14961 | -0,58588 | -0,10936 | 0,884815 | -0,3664 | 0,640725 |
| 1 | -0,15086 | -0,58662 | -0,1289 | 0,050218 | -1,24365 | 1,137553 |
| 1 | -0,15086 | -0,58662 | -0,1289 | -1,00195 | 1,140948 | -0,24117 |
| 1 | -0,15086 | -0,58662 | -0,1289 | -0,07328 | -1,2045 | 1,113903 |
| 1 | -0,15086 | -0,58662 | -0,1289 | 0,518027 | -0,77862 | 0,875107 |
| 1 | -0,15086 | -0,58662 | -0,1289 | 0,476254 | 0,209152 | 0,307761 |
| 1 | -0,15086 | -0,58662 | -0,1289 | -0,50011 | -0,67486 | 0,805834 |
| 1 | -0,15086 | -0,58662 | -0,1289 | 0,481697 | -1,53214 | 1,307256 |
| 1 | -0,14787 | -0,4075 | 2,575271 | 0,473789 | -1,45509 | 1,096785 |
| 1 | -0,15086 | -0,58662 | -0,1289 | -0,64896 | -2,16817 | 1,661526 |
| 1 | -0,1488 | -0,58647 | -0,12635 | 1,002874 | -1,84026 | 1,488731 |
| 0 | -0,15086 | -0,58662 | -0,1289 | -0,54976 | -2,05361 | 1,596718 |
| 0 | -0,07993 | 0,85233 | -0,1289 | -0,86144 | 0,623283 | -0,03311 |
| 1 | -0,15055 | -0,46357 | -0,10873 | 2,47406 | 0,137314 | 0,359706 |
| 0 | -0,15086 | -0,58662 | -0,1289 | 0,219311 | -0,30874 | 0,602562 |
| 1 | -4,45802 | -0,30534 | -0,1289 | -0,79113 | 0,420519 | 0,56021 |
| 1 | -0,15086 | -0,58662 | -0,1289 | 1,124592 | 0,083701 | 0,385953 |
| 1 | -0,15086 | -0,58662 | -0,1289 | 0,457976 | 0,635764 | 0,062725 |
| 1 | -0,15086 | -0,58662 | -0,1289 | -0,27987 | -2,16234 | 1,661702 |
| 1 | -0,15087 | -0,52489 | -0,10369 | 0,979651 | -0,39236 | 0,652771 |
| 1 | -0,34693 | 1,724792 | -0,1289 | -0,38257 | 0,757009 | -0,13117 |
| 0 | -0,15086 | -0,58662 | -0,1289 | -0,41394 | -0,94898 | 0,963996 |
| 0 | -0,87726 | 2,432851 | -0,1289 | -0,88689 | 0,994781 | -0,26416 |
| 1 | -0,15086 | -0,58662 | -0,1289 | -0,23045 | -0,26003 | 0,570312 |
| 1 | -0,15086 | -0,58662 | -0,1289 | 0,51072 | -0,13462 | 0,505402 |
| 1 | -0,15086 | -0,58662 | -0,1289 | 0,65202 | -0,05915 | 0,463436 |
| 1 | -0,30764 | 0,875406 | -0,1289 | -0,7799 | 0,079235 | 0,29985 |
| 0 | -0,15086 | -0,58662 | -0,1289 | -0,18027 | -1,74912 | 1,425479 |
| 1 | -0,15086 | -0,58662 | -0,1289 | 0,443438 | -1,16584 | 1,096648 |
| 1 | -0,15086 | -0,58662 | -0,1289 | -1,00305 | -0,70805 | 0,820088 |
| 1 | -0,15086 | -0,58662 | -0,1289 | 0,539194 | -0,4349 | 0,678025 |
| 1 | -0,14931 | -0,56699 | -0,11954 | 0,979535 | -0,10334 | 0,490098 |
| 1 | 0,412084 | 2,109458 | -0,1289 | -0,42154 | -0,093 | 0,263054 |
| 0 | -0,18403 | 2,512478 | -0,1289 | -0,86683 | 0,283682 | 0,074806 |
| 1 | -0,15086 | -0,58662 | -0,1289 | -0,48666 | -1,72963 | 1,411367 |
| 1 | -0,15086 | -0,58662 | -0,1289 | -0,97516 | -0,06071 | 0,448803 |
| 1 | -0,1502 | -0,39927 | -0,12827 | 0,107139 | -0,02054 | 0,425064 |
| 0 | -0,15585 | 2,118614 | -0,1289 | -0,76285 | 0,309101 | 0,081511 |
| 1 | -0,15086 | -0,58662 | -0,1289 | -0,78921 | -0,77495 | 0,860523 |
| 1 | -0,15086 | -0,58662 | -0,1289 | 1,3918 | -0,15212 | 0,523858 |
| 1 | -0,14895 | -0,53107 | -0,12072 | 0,824293 | -0,06331 | 0,463586 |
| 1 | -0,56801 | 0,741365 | -0,1289 | -0,92634 | -0,4284 | 0,62194 |
| 1 | -0,15086 | -0,58662 | -0,1289 | -0,19237 | 0,64185 | 0,053026 |
| 0 | 0,203058 | -0,34321 | 13,01514 | -0,2373 | -1,44803 | 0,44924 |
| 0 | -0,15066 | -0,13282 | -0,12752 | 0,890799 | 0,266711 | 0,252158 |
| 1 | -0,15086 | -0,58662 | -0,1289 | -0,35373 | -1,06037 | 1,028502 |
| 1 | -0,15086 | -0,58662 | -0,1289 | 3,261735 | 0,956948 | -0,09487 |
| 0 | 1,048024 | -0,56113 | -0,1289 | -0,89988 | -1,71089 | 1,28322 |
| 0 | -0,15086 | -0,58662 | -0,1289 | -0,99844 | 1,116678 | -0,2272 |
| 0 | -0,19698 | 2,36299 | -0,1289 | -0,72674 | 0,629797 | -0,1126 |
| 1 | -0,15088 | -0,58601 | -0,12633 | 0,447181 | -1,44831 | 1,258631 |
| 0 | -0,15086 | -0,58662 | -0,1289 | 2,328626 | 1,315354 | -0,30948 |
| 1 | -0,15086 | -0,58662 | -0,1289 | -0,6767 | -0,52178 | 0,716287 |
| 0 | 0,063996 | 0,153582 | -0,12889 | -0,57729 | 0,036918 | 0,333396 |
| 1 | -0,15086 | -0,58662 | -0,1289 | -0,52197 | 0,129326 | 0,344052 |
| 1 | -0,15086 | -0,58662 | -0,1289 | 2,420295 | 0,06648 | 0,408203 |
| 1 | -4,33079 | 3,829136 | -0,1289 | -0,95526 | 0,881826 | 0,041242 |
| 1 | -0,03089 | -0,58328 | -0,12874 | 0,031416 | -1,68884 | 1,381489 |
| 1 | -0,15086 | -0,58662 | -0,1289 | 1,416953 | -0,19511 | 0,548769 |
| 1 | -0,15086 | -0,58662 | -0,1289 | -0,56512 | 0,064431 | 0,380887 |
| 1 | -0,14903 | 0,130433 | 2,684784 | 0,451329 | -0,554 | 0,541863 |
| 1 | -0,15086 | -0,58662 | -0,1289 | -0,11482 | 1,04177 | -0,17577 |
| 0 | -0,20054 | 3,303968 | -0,1289 | -0,87717 | 0,324539 | 0,006711 |
| 1 | -0,1504 | -0,58207 | -0,12834 | 0,277712 | -0,69494 | 0,824445 |
| 1 | -0,15048 | -0,34809 | 0,132705 | -0,05128 | 0,173381 | 0,294293 |
| 0 | -0,76575 | 1,559991 | -0,1289 | -0,85841 | 0,958718 | -0,20277 |
| 0 | -1,4173 | 2,485114 | -0,12889 | -0,36109 | 0,260292 | 0,209824 |
| 1 | -0,15086 | -0,58662 | -0,1289 | -1,00182 | -0,71815 | 0,825895 |
| 0 | -0,15086 | -0,58662 | -0,1289 | -0,60127 | 2,469772 | -1,00004 |
| 1 | -0,15086 | -0,58662 | -0,1289 | -0,04842 | 0,125649 | 0,350681 |
| 1 | -0,15086 | -0,58662 | -0,1289 | -0,05152 | 0,611467 | 0,071809 |
| 1 | -0,15086 | -0,58662 | -0,1289 | -0,28015 | -1,28166 | 1,15622 |
| 0 | -0,15086 | -0,58662 | -0,1289 | -0,29657 | 1,948653 | -0,69803 |
| 1 | 4,590844 | 0,353423 | -0,1289 | -0,95864 | -0,2233 | 0,044721 |
| 1 | -0,15086 | -0,58662 | -0,1289 | -0,99652 | -0,11162 | 0,477816 |
| 1 | -0,15086 | -0,58662 | -0,1289 | -0,69912 | -0,96059 | 0,967935 |
| 1 | -0,10798 | 2,249936 | -0,03293 | 2,632721 | 1,6612 | -0,67978 |
| 1 | -0,15086 | -0,58662 | -0,1289 | 0,257506 | 0,705823 | 0,020601 |
| 1 | 2,514445 | -0,359 | -0,1289 | -0,47659 | -0,11112 | 0,220328 |
| 0 | -0,15086 | -0,58662 | -0,1289 | 2,054283 | 2,191626 | -0,81505 |
| 1 | -0,15086 | -0,58662 | -0,1289 | -0,50547 | -1,66453 | 1,373822 |
| 0 | -0,15084 | -0,36846 | -0,12637 | 0,889246 | 0,665477 | 0,036936 |
| 0 | -0,15086 | 0,738386 | -0,1289 | -0,65174 | 1,422744 | -0,47672 |
| 1 | -0,15086 | -0,58662 | -0,1289 | -0,85716 | -1,5568 | 1,308631 |
| 0 | -0,15086 | -0,58662 | -0,1289 | -0,70068 | -1,04108 | 1,014118 |
| 0 | -0,29066 | 0,142365 | -0,12889 | -0,67866 | 1,513984 | -0,48158 |
| 1 | -0,15086 | -0,58662 | -0,1289 | -0,99925 | 0,443434 | 0,159209 |
| 1 | -0,15086 | -0,58662 | -0,1289 | -0,1404 | -0,87018 | 0,921373 |
| 0 | -0,15086 | -0,58662 | -0,1289 | 1,130584 | 1,136021 | -0,21799 |
| 1 | -0,15086 | -0,58662 | -0,1289 | -0,05793 | -1,18638 | 1,103652 |
| 1 | -0,15086 | -0,58662 | -0,1289 | -0,13991 | -0,36311 | 0,630337 |
| 1 | -0,15086 | -0,58662 | -0,1289 | -1,00829 | 0,260986 | 0,263842 |
| 1 | -0,14539 | -0,57768 | -0,07469 | 1,962669 | -1,19416 | 1,123252 |
| 1 | -0,15086 | -0,58662 | -0,1289 | 0,927805 | 0,807761 | -0,03151 |
| 0 | 0,413992 | 0,21299 | -0,1289 | -0,85899 | -0,32603 | 0,502883 |
| 1 | -0,14059 | -0,56809 | -0,12588 | 1,343057 | -0,75128 | 0,865076 |
| 1 | -0,15086 | -0,58662 | -0,1289 | 1,047261 | -0,00761 | 0,437625 |
| 0 | -0,15086 | -0,58662 | -0,1289 | -0,1896 | 0,529556 | 0,117505 |
| 1 | -0,15086 | -0,58662 | -0,1289 | -1,00797 | 0,757607 | -0,0212 |
| 1 | -0,13117 | -0,58634 | -0,12851 | 0,508008 | -1,61334 | 1,352233 |
| 1 | -0,15086 | -0,58662 | -0,1289 | -0,10335 | 0,101137 | 0,364226 |
| 0 | -0,15086 | -0,58662 | -0,1289 | -0,15754 | 0,702443 | 0,01858 |
| 1 | -0,15086 | -0,58662 | -0,1289 | -0,35476 | 1,096879 | -0,2097 |
| 1 | 2,070878 | -0,48399 | -0,1289 | -0,96698 | -1,59668 | 1,117011 |
| 1 | -0,14554 | 1,387147 | -0,1289 | -0,83686 | 0,865722 | -0,19705 |
| 1 | -0,15086 | -0,58662 | -0,1289 | -0,50336 | -0,84251 | 0,902028 |
| 1 | -0,15086 | -0,58662 | -0,1289 | 0,3494 | -0,45528 | 0,68791 |
| 1 | 3,218442 | -0,42388 | -0,1289 | -0,97951 | -0,35117 | 0,291343 |
| 1 | -0,13627 | -0,52166 | -0,12465 | 1,173449 | -0,34747 | 0,628508 |
| 1 | -0,15086 | -0,58662 | -0,1289 | 2,420212 | -0,15152 | 0,533324 |
| 0 | 2,158364 | -0,22445 | -0,1289 | -0,95085 | 0,37598 | -0,03836 |
| 1 | -0,14786 | 0,088719 | -0,12035 | 2,223369 | 0,576016 | 0,073771 |
| 1 | -0,15086 | -0,58662 | -0,1289 | 0,673512 | 0,738761 | 0,005666 |
| 1 | -0,15086 | -0,58662 | -0,1289 | 1,108787 | -0,33281 | 0,624863 |
| 1 | -0,15086 | -0,58662 | -0,1289 | -0,9969 | 0,350118 | 0,212792 |
| 0 | -0,14901 | -0,27409 | -0,12605 | 1,544103 | 0,619282 | 0,064016 |
| 1 | -0,15086 | -0,58662 | -0,1289 | -1,00755 | -0,30401 | 0,588135 |
| 1 | -0,15086 | -0,58662 | -0,1289 | 0,434598 | -1,08834 | 1,052077 |
| 1 | -0,15086 | -0,58662 | -0,1289 | -0,24417 | 0,009929 | 0,415233 |
| 0 | -0,15086 | -0,58662 | -0,1289 | 0,149646 | 0,371177 | 0,211647 |
| 0 | -0,14965 | -0,40642 | -0,11979 | 1,065822 | 0,421167 | 0,180568 |
| 1 | -0,15086 | -0,58662 | -0,1289 | -0,84837 | -1,60674 | 1,337382 |
| 0 | 6,767066 | -0,10971 | -0,1289 | -0,35503 | -0,63342 | 0,109607 |
| 1 | -0,15069 | -0,16255 | -0,12482 | 0,968069 | 0,113263 | 0,342548 |
| 1 | -0,15086 | -0,58662 | -0,1289 | -0,4633 | -0,67413 | 0,805766 |
| 1 | -0,15086 | -0,58662 | -0,1289 | -0,25093 | -0,05015 | 0,449653 |
| 1 | 0,197433 | -0,45459 | -0,12887 | -0,30685 | -0,74929 | 0,810184 |
| 1 | -0,15086 | -0,58662 | -0,1289 | -0,83181 | -1,48608 | 1,268282 |
| 0 | 0,783871 | 2,609243 | -0,1289 | -0,90116 | 1,44455 | -0,68785 |
| 1 | -0,26073 | 0,467566 | -0,1289 | -0,9242 | -0,23708 | 0,499392 |
| 0 | -0,14585 | 2,561787 | -0,11834 | 2,297514 | 1,013257 | -0,32079 |
| 1 | -0,14383 | 3,19738 | -0,12868 | 1,734599 | 0,209403 | 0,098618 |
| 1 | -0,15086 | -0,58662 | -0,1289 | 0,257553 | 1,117956 | -0,21595 |
| 1 | -0,15086 | -0,58662 | -0,1289 | -1,00514 | -1,17356 | 1,087254 |
| 1 | -0,14843 | -0,57976 | -0,11697 | 1,404517 | -0,56407 | 0,759109 |
| 1 | -0,15086 | -0,58662 | -0,1289 | -0,38462 | -0,81279 | 0,886104 |
| 1 | -0,15086 | -0,58662 | -0,1289 | -0,99721 | -1,43447 | 1,237084 |
| 0 | -0,15048 | 1,158024 | -0,12873 | 0,908864 | 1,415992 | -0,48243 |
| 1 | -0,14107 | -0,51502 | -0,12427 | 1,251557 | -0,52915 | 0,733568 |
| 1 | -0,11222 | -0,5483 | -0,06003 | 1,676558 | -0,17318 | 0,528863 |
| 1 | -0,15088 | -0,57837 | -0,12875 | -0,20323 | -0,56004 | 0,742281 |
| 1 | -0,15086 | -0,58662 | -0,1289 | -0,76733 | -1,35758 | 1,195144 |
| 0 | -0,08903 | 0,214416 | -0,1289 | -0,64292 | 3,034868 | -1,3772 |
| 1 | -0,13987 | 1,726214 | -0,09928 | 1,953768 | 1,518272 | -0,56693 |
| 1 | -0,15086 | -0,58662 | -0,1289 | -0,19043 | 1,128996 | -0,22656 |
| 0 | -0,15086 | -0,58662 | -0,1289 | 0,315139 | 3,325651 | -1,48254 |
| 1 | -0,15086 | -0,58662 | -0,1289 | -0,1644 | -0,17498 | 0,522124 |
| 0 | -0,12119 | 0,098103 | -0,1092 | 1,553725 | 0,663369 | 0,013565 |
| 1 | -0,14936 | 0,642548 | -0,12723 | 2,11229 | 0,679473 | -0,01838 |
| 1 | -0,15086 | -0,58662 | -0,1289 | 0,207119 | 0,278937 | 0,265138 |
| 1 | -0,13311 | 0,298067 | -0,11817 | 2,040751 | -0,00461 | 0,391592 |
| 1 | -0,15086 | -0,58662 | -0,1289 | 1,884891 | -0,46147 | 0,70612 |
| 1 | -0,15086 | -0,58662 | -0,1289 | 0,482204 | -0,55567 | 0,746799 |
| 1 | -0,13555 | -0,50419 | -0,09108 | 1,456723 | 0,43884 | 0,176881 |
| 0 | 1,163443 | 0,491934 | -0,1289 | -0,70833 | -0,02543 | 0,245551 |
| 1 | -0,14599 | 1,487939 | -0,09234 | 1,375116 | -0,02845 | 0,329359 |
| 1 | -0,15086 | -0,58662 | -0,1289 | -0,02957 | -1,21181 | 1,118515 |
| 1 | -0,15086 | -0,58662 | -0,1289 | -0,33719 | -1,33722 | 1,18756 |
| 1 | -0,15089 | -0,5856 | -0,12722 | -0,17429 | -0,99212 | 0,990889 |
| 1 | -0,1172 | -0,57117 | -0,07737 | 2,015463 | -0,80424 | 0,897094 |
| 0 | -0,15086 | -0,58662 | -0,1289 | -0,9982 | -0,42473 | 0,657514 |
| 1 | -1,37436 | -0,41562 | -0,1289 | -0,89754 | -0,93021 | 1,052907 |
| 1 | -0,14808 | -0,55718 | -0,12381 | 1,287268 | -0,44424 | 0,68826 |
| 1 | -0,1301 | -0,53169 | -0,1261 | 2,057277 | -1,36764 | 1,222575 |
| 0 | -0,14203 | -0,21961 | -0,12865 | 1,611802 | 0,474898 | 0,143858 |
| 1 | -0,08846 | -0,24176 | 0,124825 | 2,119416 | -0,08065 | 0,449281 |
| 1 | -0,14211 | -0,58584 | -0,12536 | 1,770956 | -1,2816 | 1,174692 |
| 1 | -0,15086 | -0,58662 | -0,1289 | -0,74968 | -1,22564 | 1,119582 |
| 0 | 1,249321 | 0,304919 | -0,1289 | -0,72958 | 1,162526 | -0,43363 |
| 1 | -0,13942 | -0,53641 | 5,901521 | 1,471469 | -1,40451 | 0,892762 |
| 1 | -0,15086 | -0,58662 | -0,1289 | -0,21803 | -0,28393 | 0,584149 |
| 1 | 0,780956 | -0,31814 | -0,1289 | -0,92674 | -0,4764 | 0,585202 |
| 1 | -0,22935 | 1,98155 | -0,1289 | -0,69574 | 1,178402 | -0,40195 |
| 1 | -0,14747 | -0,5005 | -0,12714 | 1,036588 | -0,39235 | 0,65292 |
| 0 | -0,14737 | -0,40669 | -0,12528 | 1,726086 | -0,19783 | 0,54227 |
| 1 | -0,15086 | -0,58662 | -0,1289 | -0,73732 | -0,70836 | 0,822802 |
| 0 | -0,15022 | 1,133639 | -0,12886 | 0,369453 | 0,501002 | 0,038998 |
| 0 | -0,26394 | -0,53182 | -0,12888 | -0,62004 | -1,06863 | 1,03807 |
| 1 | -0,15086 | -0,58662 | -0,1289 | -1,00289 | 0,127588 | 0,340459 |
| 1 | -0,07195 | 2,31822 | -0,12888 | -0,74173 | 0,474652 | -0,03277 |
| 1 | -3,28791 | -0,53995 | -0,1289 | -0,95833 | -1,6851 | 1,671552 |
| 1 | -0,23511 | 2,768342 | -0,1289 | -0,9314 | -0,27277 | 0,383443 |
| 1 | 0,196543 | 1,313063 | -0,1289 | -0,86641 | 1,090036 | -0,35371 |
| 1 | 0,041925 | -0,52758 | -0,1289 | -0,92517 | -1,02503 | 0,981322 |
| 1 | -0,257 | 1,439516 | -0,1289 | -0,8584 | 1,352489 | -0,46928 |
| 1 | 0,981835 | 1,243649 | -0,1289 | -0,96839 | -0,9153 | 0,727014 |
| 1 | 0,213134 | -0,4581 | -0,1289 | -0,97228 | -1,05982 | 0,980806 |
| 1 | 1,137008 | -0,19149 | -0,1289 | -0,9451 | -0,37741 | 0,487584 |
| 1 | -0,15086 | -0,58662 | -0,1289 | -0,53557 | 0,576682 | 0,087155 |
| 0 | -0,20364 | 0,317184 | -0,1289 | -0,93899 | 0,731033 | -0,05299 |
| 1 | 0,151472 | -0,2248 | -0,1289 | -0,61384 | -0,66184 | 0,747972 |
| 1 | 0,263579 | 1,600054 | -0,06129 | -0,18604 | 1,651027 | -0,69607 |
| 0 | 3,81837 | -0,31767 | -0,1289 | -0,5191 | -0,10007 | 0,089401 |
| 1 | -3,06104 | -0,50185 | -0,1289 | -0,86816 | -0,65214 | 1,056126 |
| 0 | 6,470714 | 0,657812 | -0,1289 | -0,96194 | -0,3623 | -0,06881 |
| 0 | 0,367621 | 0,614807 | -0,1289 | -0,92443 | -0,23655 | 0,431831 |
| 1 | -0,63604 | -0,32946 | -0,12889 | -0,14302 | -0,02784 | 0,468209 |
| 1 | -0,05314 | 0,057276 | -0,1289 | -0,52781 | -0,3193 | 0,554874 |
| 1 | -0,41973 | -0,42378 | -0,1289 | -0,92397 | -0,68297 | 0,822072 |
| 1 | -0,15352 | -0,03527 | -0,1289 | -0,77551 | 1,106322 | -0,25099 |
| 1 | 3,0721 | -0,43074 | -0,1289 | -0,70509 | -0,59037 | 0,445321 |
| 1 | -0,01943 | 0,085586 | -0,1289 | -0,59126 | -0,66198 | 0,746156 |
| 1 | 0,2723 | 2,145856 | -0,1289 | -0,3545 | -0,06164 | 0,256633 |
| 0 | -0,0851 | 0,928754 | -0,1289 | -0,44248 | 1,250345 | -0,393 |
| 1 | 0,178179 | -0,37344 | -0,1289 | -0,96047 | -1,08573 | 0,994123 |
| 0 | -0,5251 | 1,314506 | -0,1289 | -0,93029 | 0,527644 | 0,035783 |
| 0 | -0,44544 | 0,474272 | -0,12889 | -0,54716 | 1,106687 | -0,25143 |
| 0 | -0,16654 | 0,014449 | -0,1289 | -0,90994 | 0,157528 | 0,290626 |
| 0 | -1,12896 | -0,10681 | -0,1289 | -0,88935 | 0,305908 | 0,302599 |
| 0 | -0,32086 | 0,07189 | -0,11931 | -0,25782 | 0,212542 | 0,27579 |
| 0 | 1,854911 | 2,376015 | -0,12882 | -0,16018 | 0,897204 | -0,45306 |
| 0 | -0,17932 | 1,506248 | -0,1289 | -0,79676 | -0,16415 | 0,390668 |
| 0 | 3,282205 | 2,930953 | -0,1289 | -0,78864 | 0,185037 | -0,2159 |
| 0 | 0,317779 | 1,820919 | -0,12886 | -0,10602 | 1,365034 | -0,54519 |
| 1 | -0,14921 | 1,208292 | -0,1289 | -0,88258 | 0,260616 | 0,160584 |
| 0 | 1,326915 | -0,26497 | -0,12885 | -0,16973 | 0,053409 | 0,234246 |
| 0 | -0,75991 | 3,203297 | -0,1289 | -0,82827 | 1,165811 | -0,41758 |
| 0 | -0,15086 | 2,276109 | -0,1289 | -0,82701 | 1,082235 | -0,37249 |
| 1 | -0,13255 | 2,083452 | -0,12885 | 0,233275 | 0,231378 | 0,135497 |
| 0 | -0,25699 | 2,314209 | -0,1289 | -0,815 | 0,648168 | -0,11554 |
| 1 | 0,018401 | -0,15852 | -0,12889 | -0,54769 | -0,25304 | 0,522535 |
| 1 | -0,17367 | -0,35734 | -0,1289 | -0,90535 | -1,15066 | 1,063839 |
| 1 | 3,338058 | 1,481855 | -0,1289 | -0,80566 | 0,861607 | -0,52523 |
| 0 | -0,13854 | 2,451375 | -0,1289 | -0,74045 | 0,397025 | 0,010266 |
| 1 | -0,15086 | -0,58662 | -0,1289 | -0,70677 | 0,931438 | -0,1181 |
| 1 | -0,14938 | -0,13256 | -0,1289 | -0,66227 | 2,014574 | -0,76593 |
| 0 | -0,31395 | 0,557589 | -0,12888 | -0,55818 | 1,043002 | -0,23211 |
| 1 | 0,305352 | -0,47275 | -0,1288 | -0,6388 | -0,19944 | 0,482393 |
| 0 | -0,25615 | 2,343637 | -0,1289 | -0,52978 | 0,657685 | -0,12008 |
| 1 | -0,17592 | 1,759844 | -0,1289 | -0,83559 | 0,6418 | -0,08738 |
| 0 | -0,15086 | -0,58662 | -0,1289 | 1,724969 | 0,328677 | 0,251075 |
| 0 | -0,15086 | -0,58662 | -0,1289 | -0,4599 | -0,92009 | 0,946972 |
| 0 | -0,15086 | -0,58662 | -0,1289 | 0,002579 | -0,52997 | 0,727469 |
| 0 | -0,15086 | -0,58662 | -0,1289 | 1,308619 | 1,605544 | -0,48578 |
| 0 | -0,15086 | -0,58662 | -0,1289 | -0,28445 | -1,20292 | 1,110982 |
| 0 | -0,15086 | -0,58662 | -0,1289 | -0,54419 | -0,95897 | 0,968484 |
| 0 | -0,15086 | -0,58662 | -0,1289 | -1,00353 | 0,175439 | 0,312989 |
| 0 | -0,13169 | 0,952135 | -0,11442 | 0,652073 | 1,465581 | -0,50393 |
| 0 | -0,15086 | -0,58662 | -0,1289 | 1,181518 | 0,718405 | 0,022197 |
| 0 | -0,15057 | -0,20029 | -0,12878 | 1,128255 | 0,854554 | -0,07899 |
| 0 | -0,15086 | -0,58662 | -0,1289 | -0,99511 | 0,679263 | 0,023891 |
| 0 | -0,15086 | -0,58662 | -0,1289 | 1,444307 | 0,073551 | 0,39483 |
| 0 | -0,15086 | -0,58662 | -0,1289 | -0,63126 | -1,17543 | 1,091896 |
| 0 | -0,13714 | -0,40354 | -0,06744 | 0,183195 | 0,75512 | -0,02388 |
| 0 | -0,14235 | -0,53405 | 2,614717 | 1,61669 | -1,1357 | 0,928958 |
| 0 | -0,15086 | -0,58662 | -0,1289 | -1,00716 | 2,363021 | -0,94264 |
| 0 | -0,15086 | -0,58662 | -0,1289 | -1,00756 | -0,36521 | 0,623262 |
| 0 | -0,14812 | 1,897185 | -0,12624 | 1,231523 | 1,83004 | -0,7604 |
| 0 | -0,13878 | 1,858755 | -0,09912 | 2,162423 | 1,19186 | -0,38542 |
| 0 | -0,14908 | -0,15472 | -0,11693 | 1,370311 | 1,230546 | -0,29596 |
| 0 | -0,15086 | -0,58662 | -0,1289 | -0,23471 | 1,139123 | -0,2328 |
| 0 | -0,15086 | -0,58662 | -0,12514 | 1,257163 | 1,97785 | -0,70018 |
| 0 | -0,15086 | -0,58662 | -0,1289 | -0,63126 | -0,24488 | 0,557791 |
| 0 | -0,13753 | 1,864006 | -0,128 | 2,118612 | 0,434064 | 0,050349 |
| 0 | -0,15086 | -0,58662 | -0,1289 | -1,00929 | -0,84878 | 0,900802 |
| 0 | -0,15086 | -0,28181 | -0,1289 | 2,08556 | 1,311887 | -0,32756 |
| 0 | -0,15086 | -0,58662 | -0,1289 | -0,39309 | -1,16793 | 1,089861 |
| 0 | -0,14027 | 1,595527 | -0,12882 | 1,699825 | 1,864575 | -0,75878 |
| 0 | -0,15086 | -0,58662 | -0,1289 | -1,00794 | 2,115623 | -0,80065 |
| 0 | -0,15086 | -0,5866 | -0,12695 | 0,265888 | -1,8364 | 1,479715 |
| 0 | -0,15086 | -0,58662 | -0,1289 | -0,08442 | 1,379724 | -0,36946 |
| 0 | -0,15086 | -0,58662 | -0,1289 | 0,057938 | 0,096215 | 0,368591 |
| 0 | -0,15086 | -0,58662 | -0,1289 | -0,99226 | 0,276793 | 0,254922 |
| 0 | -0,15086 | -0,58662 | -0,1289 | -0,23619 | 0,742812 | -0,00534 |
| 0 | -0,15086 | -0,58662 | -0,1289 | -1,00384 | 0,277007 | 0,254689 |
| 0 | -0,15086 | -0,58662 | -0,1289 | -1,00283 | 1,166906 | -0,25607 |
| 0 | -0,15086 | -0,58662 | -0,1289 | -0,52883 | -0,24948 | 0,561408 |
| 0 | -0,15086 | -0,58662 | -0,1289 | -0,31541 | 0,494392 | 0,136487 |
| 0 | -0,15086 | -0,58662 | -0,1289 | -1,00618 | -0,60906 | 0,763241 |
| 0 | -0,15086 | -0,58662 | -0,1289 | -0,67951 | -0,33379 | 0,608362 |
| 0 | -0,11219 | -0,51903 | -0,12479 | 2,022368 | -0,28791 | 0,60003 |

**Radscore_validation**

| Label | ClusterShade_angle0_offset7 | GLCMEntropy_AllDirection_offset7_SD | LowGreyLevelRunEmphasis_AllDirection_offset7_SD | ShortRunLowGreyLevelEmphasis_angle0_offset1 | SurfaceVolumeRatio | radscore |
| --- | --- | --- | --- | --- | --- | --- |
| 1 | -0,15086 | -0,58662 | -0,1289 | 0,319592 | 0,325857 | 0,239281 |
| 1 | 0,814886 | -0,58014 | -0,12842 | -0,80777 | -1,94475 | 1,441177 |
| 0 | 4,027711 | 3,534574 | -0,1289 | -0,76017 | 0,66581 | -0,59635 |
| 1 | -0,15086 | -0,58662 | -0,1289 | -0,08391 | 1,566447 | -0,47663 |
| 0 | -0,15086 | -0,58662 | -0,1289 | 0,380536 | 0,323134 | 0,241425 |
| 1 | -0,15086 | -0,58662 | -0,1289 | -0,46679 | -1,313 | 1,172422 |
| 1 | -0,15086 | -0,58662 | -0,1289 | -0,48024 | -1,99827 | 1,565615 |
| 0 | 0,039724 | -0,33729 | -0,12887 | -0,1873 | -0,19762 | 0,502586 |
| 0 | 0,783106 | -0,57859 | -0,1289 | -0,99584 | -1,82381 | 1,372872 |
| 1 | -0,15086 | -0,58662 | -0,1289 | -0,67424 | -0,22984 | 0,548748 |
| 0 | -0,45529 | 0,616692 | -0,1289 | -0,96914 | 0,343928 | 0,174972 |
| 0 | 1,429524 | 0,260921 | -0,1289 | -0,85946 | -0,29324 | 0,386424 |
| 0 | -0,15061 | -0,55354 | -0,12178 | 1,33795 | 0,185731 | 0,327068 |
| 0 | 0,415047 | -0,05106 | -0,1289 | -0,95704 | 0,214686 | 0,206871 |
| 1 | -0,15086 | -0,58662 | -0,1289 | 0,40997 | -0,34743 | 0,626588 |
| 0 | -0,15086 | -0,58662 | -0,1289 | -0,4936 | -0,76346 | 0,856754 |
| 1 | -0,15086 | -0,58662 | -0,1289 | 0,051588 | 1,238438 | -0,28707 |
| 1 | -0,15086 | -0,58662 | -0,1289 | -0,00871 | -0,783 | 0,872596 |
| 1 | 7,064503 | -0,48534 | -0,12878 | -0,60059 | -1,03899 | 0,334136 |
| 1 | -0,15086 | -0,58662 | -0,1289 | 0,636282 | -0,59666 | 0,771799 |
| 1 | -0,15086 | -0,58662 | -0,1289 | -0,2166 | -1,35828 | 1,2008 |
| 1 | -0,15086 | -0,58662 | -0,1289 | -1,00868 | -0,80703 | 0,876842 |
| 1 | -0,151 | -0,58564 | -0,12808 | -0,03901 | -0,58852 | 0,760589 |
| 1 | 1,431275 | 2,276109 | -0,1289 | -0,81323 | 2,088022 | -1,0974 |
| 0 | -0,15086 | -0,58662 | -0,1289 | -0,44902 | 1,984693 | -0,72017 |
| 0 | -0,14199 | 0,719159 | -0,1289 | 0,456426 | 1,107782 | -0,28507 |
| 1 | 1,492375 | 0,444137 | -0,1289 | -0,82247 | 1,846965 | -0,85816 |
| 1 | -0,15086 | -0,58662 | -0,1289 | -1,0067 | -0,83757 | 0,894394 |
| 1 | -0,15086 | -0,58662 | -0,1289 | 0,589554 | 0,337746 | 0,235033 |
| 1 | -0,15086 | -0,58662 | -0,1289 | -0,34124 | -2,11684 | 1,634997 |
| 1 | -0,36343 | 1,002322 | -0,1289 | 0,474016 | 1,693819 | -0,61708 |
| 0 | -1,43771 | 1,132501 | -0,1289 | -0,66738 | 1,417589 | -0,37668 |
| 1 | 0,055386 | 1,836374 | -0,12874 | -0,17744 | -0,08947 | 0,312561 |
| 1 | -0,15086 | -0,58662 | -0,1289 | -0,51726 | -0,09804 | 0,474594 |
| 1 | -0,15086 | -0,58662 | -0,1289 | -0,37079 | -1,58371 | 1,328719 |
| 1 | -0,14523 | -0,26685 | -0,12577 | 1,95399 | 0,177218 | 0,320867 |
| 1 | -0,15106 | 2,079449 | -0,12863 | 1,289725 | 0,703569 | -0,12349 |
| 0 | -0,41203 | 2,219951 | -0,12889 | -0,44677 | 0,962852 | -0,27268 |
| 1 | -0,14847 | -0,58122 | -0,12771 | 0,965959 | -0,99917 | 1,005361 |
| 1 | -0,15052 | -0,55581 | 9,212657 | 0,480311 | -1,22171 | 0,590195 |
| 0 | -0,15086 | -0,58662 | -0,1289 | -0,34433 | 0,733414 | -9,79E-04 |
| 1 | -0,13785 | -0,58467 | -0,12665 | 1,352146 | -0,59471 | 0,776052 |
| 1 | -0,15086 | -0,58662 | -0,1289 | -0,18417 | -0,67895 | 0,8112 |
| 1 | -0,15086 | -0,58662 | -0,1289 | 0,124996 | -0,81238 | 0,890732 |
| 0 | -0,15086 | -0,58662 | -0,1289 | -1,00488 | 0,292213 | 0,245951 |
| 1 | -0,15086 | -0,58662 | -0,1289 | -0,63366 | -0,6171 | 0,771407 |
| 1 | 3,288837 | -0,33552 | -0,1289 | -0,97271 | -0,926 | 0,609623 |
| 1 | -0,15086 | -0,58662 | -0,1289 | 1,216317 | -0,01978 | 0,446222 |
| 1 | -0,14706 | -0,5852 | -0,11667 | 1,817706 | -1,06703 | 1,051905 |
| 1 | -0,15086 | -0,58662 | -0,1289 | -0,9981 | 0,019641 | 0,402463 |
| 1 | -0,1425 | -0,35828 | -0,11946 | 1,121464 | 0,208933 | 0,299424 |
| 0 | -0,16736 | 0,327226 | -0,1289 | 0,049109 | 2,055358 | -0,80765 |
| 1 | -0,15086 | -0,58662 | -0,1289 | 0,381071 | 1,350527 | -0,34826 |
| 1 | -0,15086 | -0,58662 | -0,1289 | 0,463116 | -0,34841 | 0,627656 |
| 1 | -0,15086 | -0,58662 | -0,1289 | 0,576422 | -0,16595 | 0,524013 |
| 1 | -0,11844 | 0,101956 | -0,02978 | 0,337707 | -0,1397 | 0,457846 |
| 1 | -0,10321 | -0,5862 | -0,09234 | 4,213256 | -1,92685 | 1,562841 |
| 0 | 1,056584 | -0,31583 | -0,1289 | -0,9116 | -0,51985 | 0,58441 |
| 0 | 0,036907 | 1,535005 | -0,1289 | -0,94535 | 0,145402 | 0,189708 |
| 0 | -0,16548 | 0,677836 | -0,12884 | -0,1595 | 0,357673 | 0,144181 |
| 1 | -0,15086 | -0,58662 | -0,1289 | 0,973742 | 1,096742 | -0,19694 |
| 1 | 2,285968 | -0,55357 | -0,1289 | -0,97998 | -1,69188 | 1,155494 |
| 1 | 7,95001 | -0,57378 | -0,1289 | -0,98002 | -1,90531 | 0,750212 |
| 1 | -0,12223 | -0,58637 | -0,1212 | 1,921548 | -1,76496 | 1,451498 |
| 0 | -0,14603 | -0,52 | -0,11855 | 1,826052 | 0,017365 | 0,425796 |
| 1 | -0,15086 | -0,58662 | -0,1289 | -0,46239 | 0,635737 | 0,053957 |
| 0 | -0,15086 | -0,58662 | -0,1289 | 1,74541 | 3,789882 | -1,73534 |
| 0 | -0,12674 | 2,578193 | -0,1289 | -0,503 | 0,95776 | -0,3178 |
| 1 | -0,14819 | 0,117586 | -0,12627 | 1,421425 | 0,747935 | -0,03387 |
| 1 | 1,135169 | -0,30675 | -0,1289 | -0,4436 | 0,058884 | 0,248833 |
| 1 | -0,13652 | -0,5442 | -0,1258 | 1,085836 | -0,46302 | 0,695397 |
| 1 | -0,15082 | -0,53942 | -0,12819 | -0,09147 | -0,37439 | 0,634485 |
| 0 | -0,15153 | -0,56804 | -0,12083 | 0,888467 | -1,01392 | 1,012213 |
| 1 | -0,15086 | -0,58662 | -0,1289 | 0,451781 | -0,70447 | 0,831914 |
| 1 | -0,15089 | 0,348835 | -0,12591 | 0,371807 | 0,426838 | 0,12718 |
| 0 | 0,419855 | -0,07744 | -0,1289 | -0,33034 | 2,801704 | -1,27092 |
| 0 | -0,14689 | 0,68392 | -0,12652 | 1,294086 | 0,031092 | 0,343276 |
| 1 | -0,13113 | -0,57575 | -0,12394 | 1,193511 | -0,48861 | 0,712336 |
| 1 | 1,342339 | -0,55663 | -0,1289 | -0,98512 | -0,18434 | 0,378473 |
| 0 | -0,15086 | -0,51041 | -0,1289 | -0,80879 | 1,847194 | -0,64912 |
| 0 | -0,15086 | -0,28181 | -0,1289 | -0,69027 | 0,562083 | 0,076309 |
| 1 | -0,16478 | 1,449688 | -0,1289 | -0,70716 | 0,964088 | -0,25411 |
| 0 | 0,54774 | 2,560958 | -0,1289 | -0,72635 | 0,547777 | -0,1466 |
| 1 | 0,753718 | -0,30675 | -0,1289 | -0,88153 | -0,21081 | 0,435074 |
| 0 | -0,15086 | 0,693389 | -0,1289 | -0,26107 | 0,635107 | -0,01829 |
| 1 | 3,327742 | 1,94907 | -0,1289 | -0,57267 | 1,012071 | -0,63561 |
| 1 | 1,378991 | -0,31558 | -0,1289 | -0,61316 | -0,66459 | 0,640209 |
| 0 | -0,04833 | -0,26258 | -0,1289 | -0,93724 | -0,37958 | 0,603738 |
| 0 | -0,25716 | 3,17732 | -0,1289 | -0,32816 | -0,19581 | 0,323277 |
| 1 | 0,286727 | 2,322585 | -0,12888 | -0,14958 | 0,431217 | -0,03594 |
| 0 | -0,46927 | 1,547502 | -0,1289 | -0,22796 | 1,182097 | -0,35193 |
| 1 | 0,237983 | -0,54342 | -0,1289 | -0,97561 | -1,22721 | 1,079497 |
| 1 | 0,344229 | -0,52133 | -0,12885 | -0,29529 | -0,75347 | 0,802869 |
| 1 | 1,874842 | -0,48675 | -0,12889 | -0,51564 | -0,83348 | 0,701737 |
| 0 | -0,15086 | -0,28181 | -0,1289 | 1,081841 | 2,832133 | -1,20971 |
| 1 | -0,10849 | 1,064372 | -0,1289 | -0,47801 | 1,003768 | -0,25752 |
| 1 | 2,548612 | 0,380343 | -0,1289 | -0,67808 | 0,547863 | -0,20607 |
| 1 | -0,77917 | -0,42649 | -0,1289 | -0,76044 | -0,04215 | 0,489548 |
| 0 | 0,148848 | 0,132485 | -0,1289 | -0,81666 | 0,163924 | 0,251519 |
| 1 | 2,758124 | 0,458775 | -0,1289 | -0,72997 | -0,54716 | 0,397805 |
| 1 | -0,15086 | 0,254277 | -0,1289 | -0,11483 | 1,589759 | -0,53927 |
| 0 | -0,19846 | 2,204772 | -0,1289 | -0,73553 | 0,703939 | -0,14589 |
| 1 | -0,15086 | -0,58662 | -0,1289 | 1,695993 | 1,290628 | -0,30133 |
| 0 | -0,15086 | -0,58662 | -0,1289 | -0,48065 | 1,375801 | -0,37099 |
| 0 | 4,06025 | -0,42762 | -0,1289 | -0,99183 | -0,67725 | 0,399983 |
| 1 | -0,11426 | 3,853846 | -0,1289 | -0,8596 | -0,61009 | 0,503247 |
| 0 | -1,14707 | 1,006422 | -0,1289 | -0,63817 | 3,924416 | -1,83504 |
| 0 | -0,15086 | -0,58662 | -0,1289 | 0,194879 | 1,186568 | -0,25593 |
| 0 | -0,13159 | -0,58327 | -0,12003 | 1,808944 | -1,87526 | 1,514356 |
| 0 | -0,15086 | -0,58662 | -0,1289 | -0,26828 | 0,672758 | 0,034561 |
| 0 | -0,15277 | 1,887212 | -0,12681 | 1,324718 | 2,758867 | -1,29158 |
| 0 | -0,15086 | -0,58662 | -0,1289 | -1,00043 | 0,307463 | 0,23724 |
| 0 | -0,15086 | -0,58662 | -0,1289 | 0,312117 | 0,196424 | 0,3135 |
| 0 | -0,14984 | -0,54189 | -0,12794 | 1,344613 | -0,51621 | 0,729625 |
| 0 | -0,14992 | 0,070529 | -0,1281 | 1,059359 | 1,585516 | -0,51506 |
| 0 | -0,15086 | -0,58662 | -0,1289 | 0,72794 | -0,13256 | 0,506292 |
| 0 | -0,15086 | -0,58662 | -0,1289 | -1,00497 | 2,519406 | -1,03238 |
| 0 | -0,15086 | -0,58662 | -0,1289 | 0,539876 | -0,17566 | 0,529239 |
